# Small molecules targeting the PD-1/PD-L1 axis for cancer immunotherapy

**DOI:** 10.7150/thno.130935

**Published:** 2026-04-08

**Authors:** Jia-Yi Yin, Hui-Min Liu, Shao-long Li, Xin-Qian Ji, Jun-Jie Wang, Meng-Jie Fu, Cong-Jun Liu, Ning Wang, Guo-Liang Lu, Yan Li, Hong-Min Liu, Yi-Chao Zheng, Xing-Jie Dai, Ying Liu

**Affiliations:** 1 Department of Pharmacy, Henan Province Engineering Research Center of Application & Translation of Precision Clinical Pharmacy, the First Affiliated Hospital of Zhengzhou University, Zhengzhou 450052, China.; 2 Key Laboratory of Advanced Drug Preparation Technologies, Ministry of Education, China; State Key Laboratory of Metabolic Dysregulation & Prevention and Treatment of Esophageal Cancer; Key Laboratory of Henan Province for Drug Quality and Evaluation; Institute of Drug Discovery and Development; School of Pharmaceutical Sciences, Zhengzhou University, Zhengzhou 450001, China.; 3 Department of Orthopaedics, Qilu Hospital, Shandong University Centre for Orthopaedics, Advanced Medical Research Institute, Cheeloo College of Medicine, Shandong University, Shandong, 250012, China.; 4 School of Food and Health Engineering, Zhengzhou University of Technology, Zhengzhou 450000, China; 5 School of Chinese Medicine, University of Hong Kong, 3, Sasson Road, Pokfulam, Hong Kong.; 6 Auckland Cancer Society Research Centre, Faculty of Medical and Health Sciences, The University of Auckland, Private Bag 92019, Auckland 1142, New Zealand.; 7 Maurice Wilkins Centre, The University of Auckland, Private Bag 92019, Auckland 1142, New Zealand.; 8 Department of Biomedicine and Medical Diagnostics, School of Science, Auckland University of Technology. 34 St. Paul Street, Auckland 1010, New Zealand.

**Keywords:** PD-1, PD-L1, inhibitors, degraders, dual-target inhibitors, immunotherapy, anticancer

## Abstract

PD-1/PD-L1 pathway, a key immune checkpoint, triggers T-cell exhaustion via binding and aiding tumor immune evasion. Although several anti-PD-1/PD-L1 monoclonal antibodies (mAbs) have been granted food and drug administration (FDA) approval, their high cost, poor oral bioavailability, and potential immunogenicity have led to a shift in research toward small molecules. This review summarizes the structure and function of PD-1/PD-L1 and, based on the PD-1/PD-L1 signaling process, focuses on three major classes of related compounds: small molecule inhibitors inducing PD-L1 dimerization or blocking PD-1/PD-L1 binding; PD-L1 degraders (e.g., Proteolysis-targeting chimeras (PROTACs) and Lysosome-targeting chimeras (LYTACs)) via the ubiquitin-proteasome or lysosomal pathway, overcoming membrane protein targeting; and dual-target inhibitors that enhance therapeutic efficacy by exerting synergistic effects. While small molecule drugs have advantages over monoclonal antibodies, including oral administration and reduced immunogenicity, they face drug resistance and toxicity challenges. This review aims to provide insights into the discovery of safe and effective antitumor immunotherapeutic agents.

## 1. Introduction

Cancer continues to be the second leading cause of mortality worldwide, underscoring the critical demand for safer and more potent therapeutic interventions 1. Malignant cells evade immune surveillance through diverse immunosuppressive mechanisms 2. Immune checkpoint inhibitors constitute a promising therapeutic paradigm to amplify antitumor immunity by targeting these regulatory molecules 3. Since the first cytotoxic T-lymphocyte-associated antigen 4 (CTLA-4) monoclonal antibody, ipilimumab, was approved in 2011, tumor immunotherapy, particularly Immune Checkpoint Therapy (ICT), has undergone remarkable progress and rapid clinical translation 4. To date, many ICT monoclonal antibody drugs have been authorized globally for the therapeutic intervention of more than 20 tumor types, encompassing over 100 indications 5. The durable clinical responses achieved using ICT have provided unprecedented therapeutic benefits to patients 6. However, as protein-based therapeutics, monoclonal antibodies are associated with inherent drawbacks, including strong immunogenicity, slow metabolic clearance, poor tissue penetration, high production costs. These factors collectively restrict the broader clinical applications of ICT 7.

PD-1 and PD-L1 represent the most extensively characterized immune checkpoints 8. Upon ligand binding, PD-1 initiates inhibitory signal transduction via its two key cytoplasmic motifs, namely the immunoreceptor tyrosine-based switch motif (ITSM) and the immunoreceptor tyrosine-based inhibitory motif (ITIM). PD-L1, also known as CD274 or B7-H1, is a member of the B7 family and a type I transmembrane glycoprotein, serving as the primary ligand for the PD-1 9. PD-L1 is highly expressed in various malignant tumors, such as lung cancer, gastric cancer, liver cancer and so on. 10. PD-1 engagement with PD-L1 induces T-cell exhaustion, which in turn permits tumor cells to elude immune surveillance 11. However, immune checkpoint therapy targeting PD-1/PD-L1 also has the aforementioned limitations 12, 13. These limitations have spurred the development of small molecule inhibitors, which offer several significant advantages over monoclonal antibodies, such as enhanced tumor penetration, minimal immunogenicity, reduced manufacturing costs, and the feasibility of oral administration (Table 1). Their favorable pharmacokinetic properties make them compelling candidates for augmenting the scope and efficacy of cancer immunotherapy 14. Currently, most PD-1/PD-L1 inhibitors in clinical stages are in phase I, and many are based on a biphenyl core structure. The sluggish clinical progress has prompted researchers to shift their focus to PROTACs, LYTACs, dual-target inhibitors and other alternative modalities, aiming to provide novel insights for PD-1/PD-L1 drug development 15.

Existing reviews on the PD-1/PD-L1 pathway have predominantly focus on monoclonal antibodies, with small molecule drugs often mentioned only peripherally or restricted to a single class of compounds 15-19. In contrast, this review provides a comprehensive and systematic focus on small molecule drugs targeting the PD-1/PD-L1 axis. It delineates the structural characteristics and biological functions of this pathway, and traces the evolutionary progression of both small molecule inhibitors and degraders. A particular emphasis is placed on the recent advancements in dual-target inhibitors, which co-inhibit the PD-1/PD-L1 pathway alongside other key signaling cascades to enhance antitumor efficacy. By integrating discussions on mechanisms of action, structural classification, drug design strategies, and clinical challenges, this review aims to consolidate the core breakthroughs in the field and outline future directions for the development of next-generation PD-1/PD-L1-targeted small molecule therapeutics.

## 2. Structure and functions of PD-1/PD-L1

### 2.1. Structure of PD-1/PD-L1

PD-1 functions as a type I transmembrane glycoprotein (approximately 50-55 kDa) that is critical to regulating immune tolerance, especially in T cells 9. It comprises an extracellular IgV-like domain, a transmembrane region, and an intracellular tail harboring critical signaling motifs 11. The extracellular domain exhibits an α/β-sandwich immunoglobulin fold, consisting of nine β-strands linked by eight loops—notably, a flexible N-terminal loop is indispensable for ligand engagement. The structural integrity is maintained by a disulfide bond linking the cysteine residues at positions 54 and 123 20. The intracellular domain encompasses two key signaling motifs, namely an ITSM and ITIM, which are crucial for transducing inhibitory signals 21. PD-1 shares a high degree of structural similarity with CD28 and CTLA-4, suggesting that they may utilize the same class of ligands, such as members of the B7 or MHC class families 22.

PD-L1, the most canonical ligand for PD-1, is a 290-amino-acid type I transmembrane protein of ~33 kDa belonging to the B7 family of immunoregulatory molecules. 9. It plays a critical role in modulating T-cell activation and immune tolerance 23. Its structure consists of an N-terminal signal peptide, an IgV-like domain containing N-linked glycosylation sites, an IgC-like domain conferring extracellular stability, a transmembrane domain, and a cytoplasmic tail featuring s-palmitoylation sites. N-linked glycosylation within these domains is essential for regulating PD-L1 expression and function activity 24, 25.

**Figure 1:**
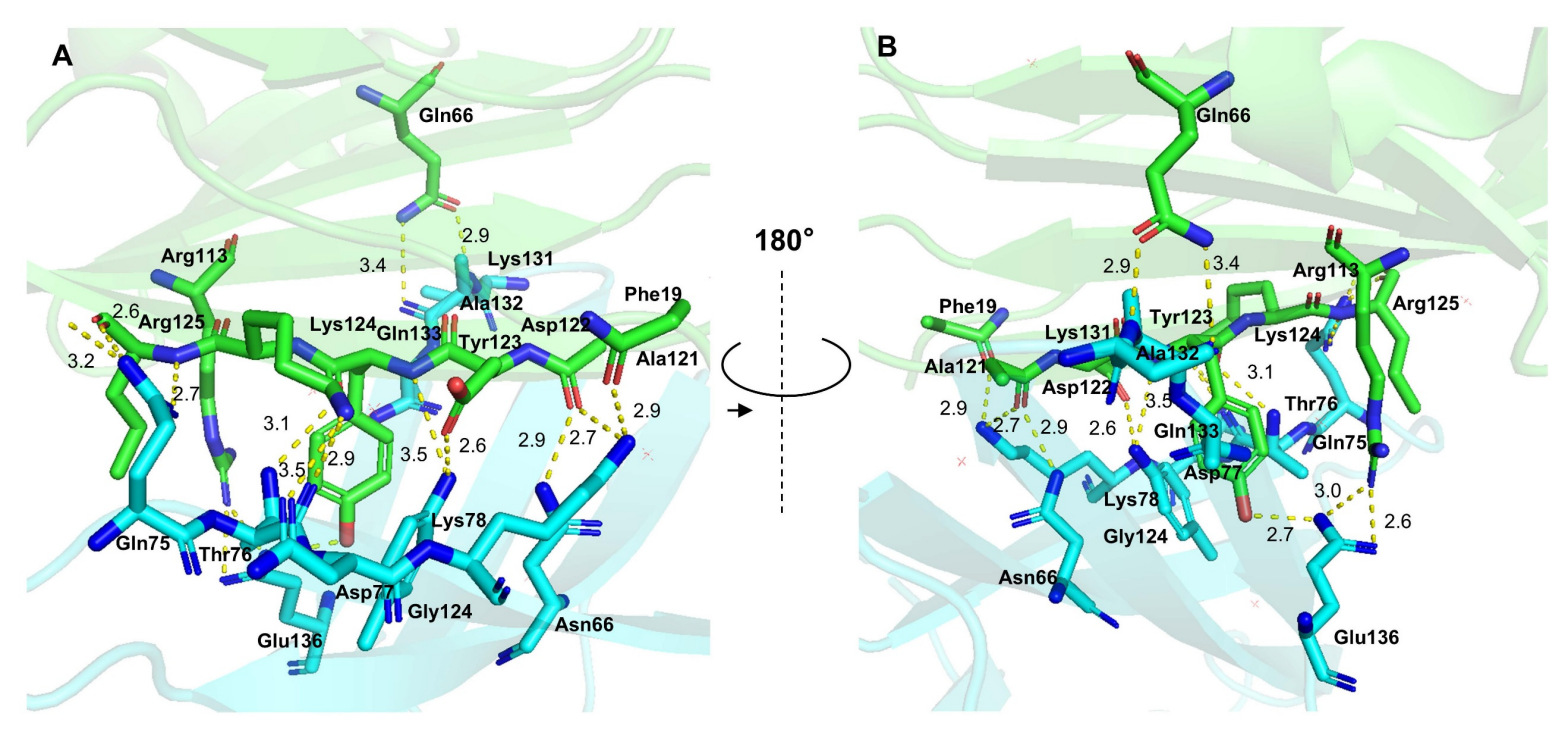
Ribbon representation of human PD-1 (blue) and human PD-L1 (green) complex (PDB ID: 4ZQK). (**A**) Front view. (**B**) Back view. Yellow dashed lines indicate hydrogen bonds stabilizing the interaction. Sticks represent the interacting residues.

The PD-1/PD-L1 combination is primarily mediated by hydrophobic residues localized in the C, C', F, and G strands of both proteins, which form a central component critical for mediating immunosuppressive effects (Figure 1) 26. Targeting and disrupting this protein-protein binding interface serve as a primary approach for small molecule inhibitors designed to block the PD-1/PD-L1 immune checkpoint 27.

### 2.2. Functions of PD-1/PD-L1

PD-1 is mainly expressed on the surface of activated immune cells, including T cells, B cells, natural killer (NK) cells, macrophages, dendritic cells (DCs), and monocytes, especially on tumor-specific T cells and functionally exhausted T cells, PD-1 is highly expressed 28-30. Upon PD-L1 association with PD-1, the tyrosine residues within the ITIM and ITSM motifs of PD-1 are phosphorylated by specific kinases, such as Lck or Src family kinases in T cells, or Lyn in B cells. This phosphorylation event promotes the association of Src homology 2 (SH2) domain-containing tyrosine phosphatase 2 (SHP2) with PD-1. Subsequently, SHP2 mediates the dephosphorylation of several critical downstream signaling molecules, including ZAP70, PI3K, and Ras, thereby suppressing signaling through the T cell receptor (TCR) and the co-stimulatory receptor CD28 11. The PD-1 signaling pathway further inhibits important T cell activation pathways such as PI3K/AKT and RAS-ERK1/2 31. The cumulative effect of this series of inhibitory signals is comprehensive, resulting in restricted T cell proliferation, reduced secretion of effector cytokines (such as IL-2 and IFN-γ), decreased cytotoxicity, and ultimately leading T cells to enter a state of functional impairment or “exhaustion” 32.

**Figure 2:**
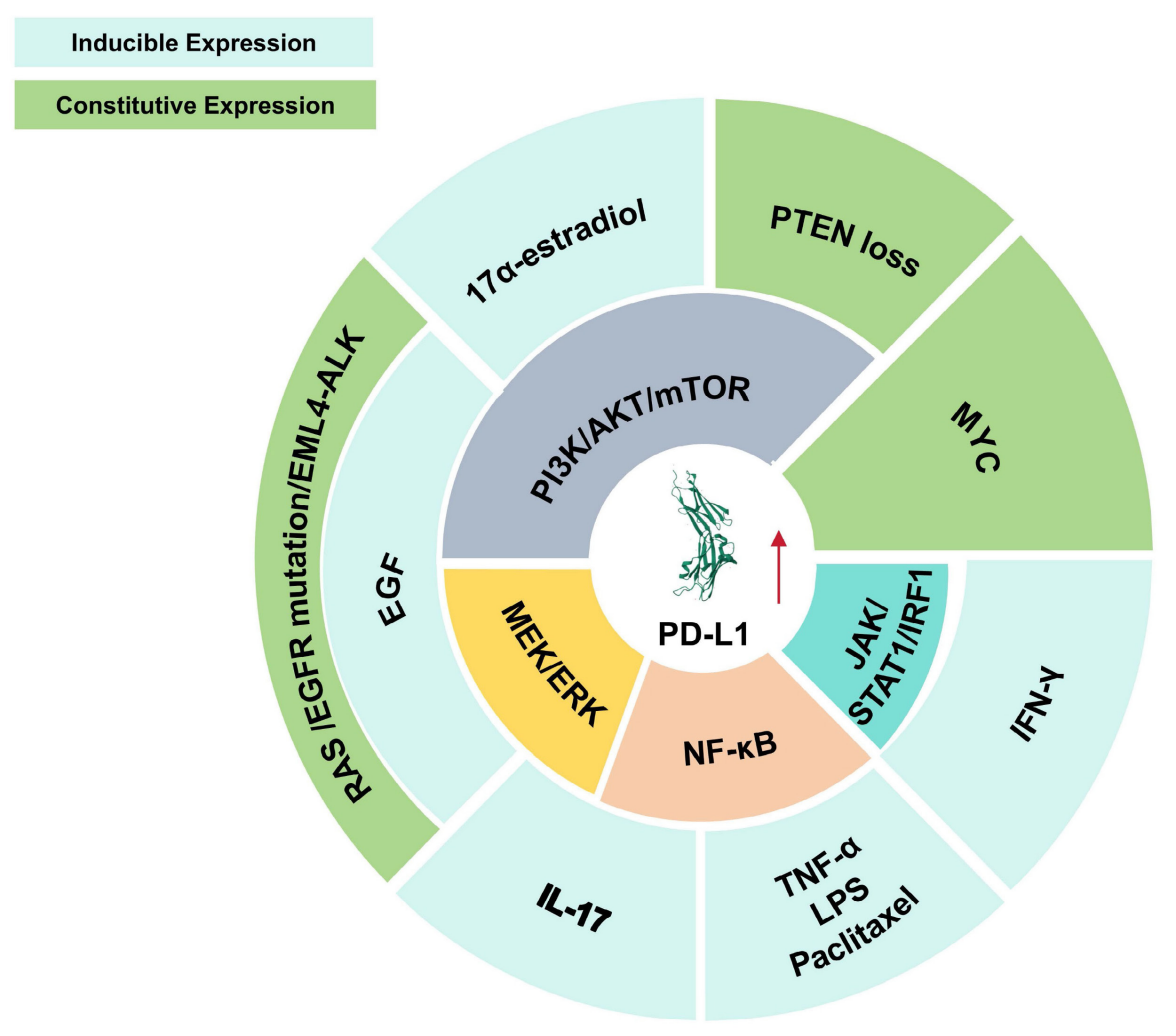
Mechanisms governing PD-L1 expression in cancer cells. PD-L1 expression in cancer cells is regulated through both inducible and constitutive mechanisms. Extrinsic stimuli, including EGF, 17α-estradiol, IFN-γ, TNF-α, IL-17, lipopolysaccharide, and paclitaxel, can enhance PD-L1 expression through multiple signaling cascades, such as PI3K/AKT/mTOR, MEK/ERK, NF-κB, and JAK/STAT1/IRF1. In parallel, constitutive PD-L1 upregulation may arise from oncogenic alterations and tumor-intrinsic events, including PTEN loss, RAS or EGFR activation, EML4-ALK rearrangement, and MYC-driven transcriptional regulation. Together, these regulatory inputs promote persistent PD-L1 expression in tumor cells and contribute to immune escape within the tumor microenvironment. The outer ring indicates upstream stimuli or genetic events, and the inner ring indicates intracellular pathways. Red arrows indicate upregulation.

PD-L1 is widely expressed across various cancer types 33. Its expression is induced by extrinsic stimuli (e.g., 17α-estradiol induces PD-L1 expression by activating the PI3K/AKT signaling pathway. Similarly, epidermal growth factor (EGF) upregulates PD-L1 by stimulating both the PI3K/AKT/mTOR and MEK/ERK pathways. Interleukin-17 (IL-17) enhances PD-L1 expression via the MEK/ERK and NF-κB signaling axes. Other stimuli, including tumor necrosis factor-alpha (TNF-α), lipopolysaccharide (LPS), and paclitaxel, also elevate PD-L1 levels by activating the NF-κB pathway. Additionally, interferon-gamma (IFN-γ) promotes PD-L1 expression through the JAK/STAT1/IRF1 signaling cascade.) or undergoes constitutive upregulation via oncogenic driver mutations and epigenetic alterations in cancer cells (e.g., phosphatase and tensin homolog (PTEN) functional impairment or loss activates the PI3K/AKT/mTOR signaling cascade, thereby enhancing PD-L1 expression. Genetic alterations in rat sarcoma virus (RAS) and epidermal growth factor receptor (EGFR), as well as EML4-ALK chromosomal translocations, concurrently activate both the PI3K/AKT/mTOR and MEK/ERK pathways, ultimately leading to PD-L1 upregulation. Furthermore, the MYC oncogene directly contributes to and promotes PD-L1 expression) (Figure 2) 34. Besides binding to PD-1, PD-L1 has also been demonstrated to bind to B7-1 (CD80), inhibiting T cell costimulatory signaling by displacing CD80 from CD28 and further restricting T cell activation 35, 36. In addition to indirectly promoting tumor growth and survival by inhibiting immunity through receptor binding, the intracellular domain of PD-L1 itself also facilitates tumor growth and metastasis via ligand-independent signaling mechanisms 37.

Within the TME, tumor cells frequently exploit the PD-1/PD-L1 pathway to suppress T cell function and evade immune monitoring 38. The interaction between PD-L1 and PD-1 induces phosphatase activation that attenuates TCR signaling, thereby impairing T cell activity and facilitating immune evasion 39, 40. To establish this immunosuppressive state, tumors upregulate PD-L1 through diverse molecular strategies, ranging from gene amplification and epigenetic changes to the activation of oncogenic pathways and alterations at the transcription and post-translational levels 41. Given its central role, disrupting the PD-1/PD-L1 interaction has emerged as a powerful therapeutic approach to reinvigorate T-cell immunity and treat a wide array of malignant tumors 42.

### 2.3. Downstream molecular mechanisms of PD-1/PD-L1-mediated immunosuppression

During T cell activation, several canonical signaling pathways are engaged. Classical TCR signaling is initiated upon antigen recognition, which triggers activation of the downstream tyrosine kinase ZAP70 through the CD3ζ chain. CD3ζ and ZAP70 are widely regarded as central “switches” in proximal TCR signaling 43, 44. Activated ZAP70 subsequently phosphorylates the adaptor protein linker for activation of T cells (LAT), thereby initiating multiple critical downstream signaling cascades. (i) The NFAT signaling axis represents the most well-established pathway downstream of LAT. Phosphorylated LAT recruits phospholipase Cγ1 (PLCγ1), which catalyzes the hydrolysis of phosphatidylinositol 4,5-bisphosphate (PIP_2_) into diacylglycerol (DAG) and inositol trisphosphate (IP_3_) 45. IP_3_ triggers intracellular Ca^2+^ release and activates calcineurin 46, which subsequently promotes the nuclear translocation of NFAT transcription factors 47. (ii) In parallel, LAT recruit the adaptor protein Grb2, which associates with SOS to activate the Ras-Raf-MEK-ERK signaling cascade (the MAPK pathway). This cascade ultimately induces the formation of the AP-1 transcription factor complex 48. (iii) Moreover, DAG generated from PLCγ1-medisted PIP_2_ hydrolysis activates protein kinase Cθ (PKCθ) 49, which stimulates the CARMA1-BCL10-MALT1 (CBM) complex, this leads to IKK activation and subsequent induction of the NF-κB signaling pathway 50. (iv) Beyond these classical pathways, T cell activation also engages the PI3K/AKT/mTOR signaling axis. Although this pathway is typically associated with TCR signaling, it is prominently driven by CD28-mediated costimulation. Mechanistically, phosphorylated CD28 recruits PI3K, which catalyzes the conversion of PIP_2_ to PIP_3_, thereby activating AKT and subsequently mTORC1. Notably, this pathway represents one of the major targets of PD-1/PD-L1 immune checkpoint-mediated inhibition 51, 52.

**Figure 3:**
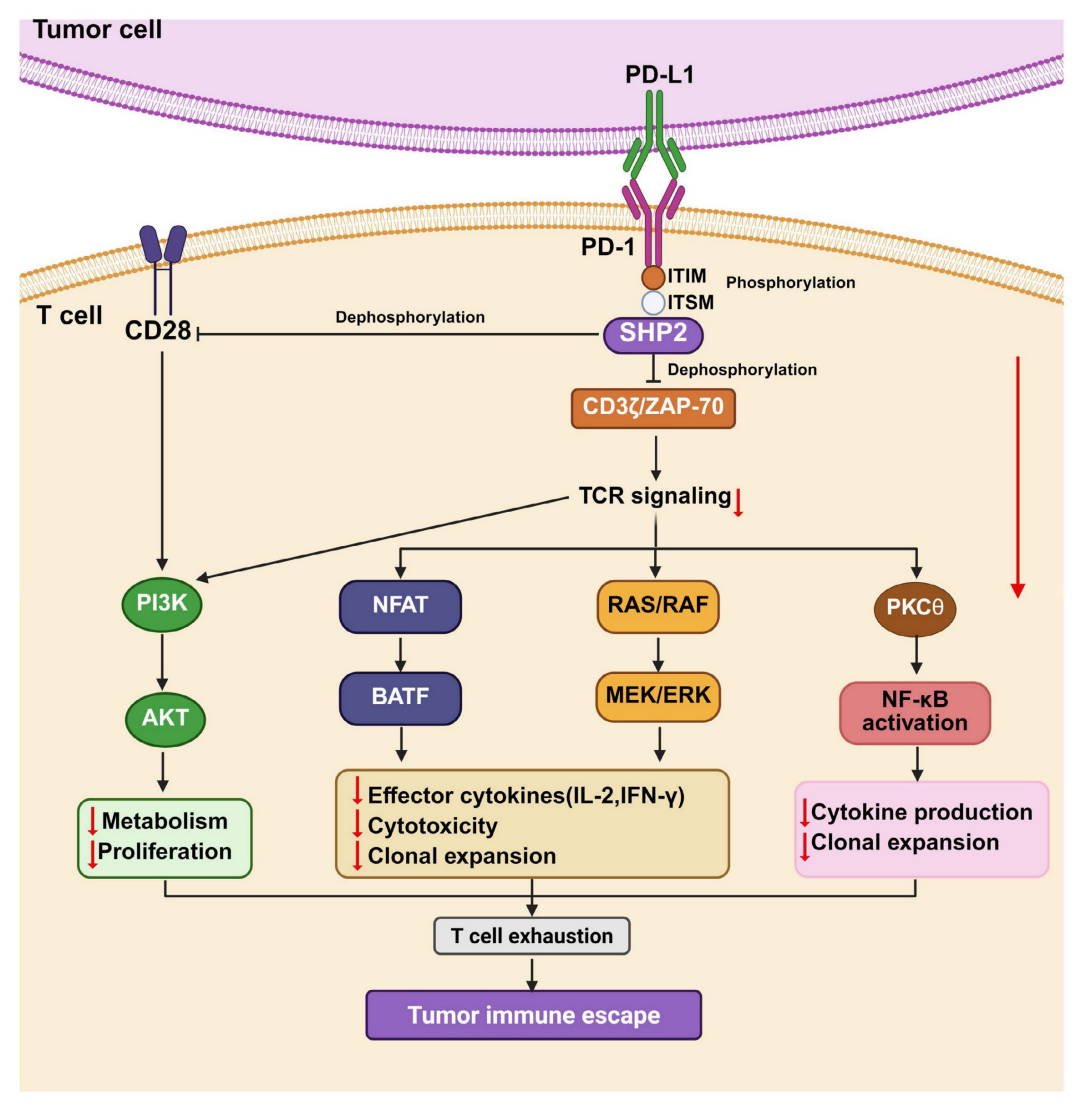
Downstream molecular mechanisms of PD-1/PD-L1-mediated immunosuppression. Upon PD-L1 binding, PD-1 undergoes cytoplasmic phosphorylation and recruits the phosphatase SHP2. Activated SHP2 dampens proximal TCR/CD28 signaling by dephosphorylating key signaling molecules, including CD3ζ, ZAP-70, and CD28, thereby weakening LAT-dependent signal propagation. As a consequence, multiple effector pathways, such as PI3K/AKT/mTOR, NFAT, Ras/RAF/MEK/ERK, and NF-κB, are coordinately suppressed. This signaling blockade limits T-cell activation, proliferation, cytokine production, and cytotoxic function, ultimately driving T-cell exhaustion and facilitating tumor immune evasion. Black arrows indicate activation, and T-shaped lines indicate inhibition. Red arrows denote changes in expression levels, with downward arrows indicating downregulation or inhibition.

The canonical inhibitory function of the PD-1/PD-L1 pathway is primarily mediated by the recruitment and activation of SHP2 53. As described above, upon PD-L1 engagement, tyrosine residues within the ITIM and ITSM motifs of PD-1 are phosphorylated by specific kinases, leading to the recruitment of SHP2 (Figure 3). Once activated, SHP2 dephosphorylates multiple key downstream signaling molecules, including ZAP70, PI3K, and Ras, thereby suppressing signal transduction from both the TCR complex and the CD28 costimulatory receptor (Figure 3). Specifically, SHP2-mediated dephosphorylate of CD28 inhibits the PI3K/AKT/mTOR pathway 52, 54, thereby impairing T cell metabolic activation and proliferation. Concurrently, SHP2 promotes the dephosphorylation of ZAP70 and CD3ζ, reducing their interaction 55, and leading to diminished phosphorylation of LAT 56. As LAT serves as a key signaling hub that orchestrates the NFAT, MAPK, and NF-κB pathways, its inactivation results in broad suppression of these downstream effector cascades, ultimately attenuating T cell activation and functional responses.

The binding of PD-1 and PD-L1 activates multiple intracellular signal transduction cascades that suppress T cell function. Upon PD-1/PD-L1 ligation, the tyrosine phosphatase SHP2 is recruited to the dephosphorylated ITSM of PD-1, where it dephosphorylates proximal signaling molecules, such as ZAP70/CD3ζ, thereby attenuating TCR signaling 57. These events lead to the downregulation of several signaling pathways, such as PI3KAKT/mTOR, NF-κB, and Ras-MAPK axes, ultimately inhibiting T cell activation, suppressing clonal proliferation, reducing proinflammatory cytokines, and promoting immune evasion. The functional consequences of PD-1/PD-L1 signaling extend beyond T cell modulation and vary across cancer types. In non-small cell lung carcinoma (NSCLC), the EML4-ALK fusion oncogene drives PD-L1 overexpression through activation of the MEK-ERK and PI3K-AKT pathways. Meanwhile, in diffuse large B-cell lymphoma (DLBCL), PD-1 engagement directly triggers AKT/mTOR signaling in malignant cells, a mechanism associated with poor clinical outcomes 58. A comprehensive understanding of these signaling networks is essential for the rational design of effective therapeutic strategies. For instance, combining PD-1/PD-L1 immune checkpoint blockade with inhibitors targeting the MEK-ERK or PI3K-AKT pathways holds promise for synergistically enhancing antitumor immunity. Furthermore, targeting key nodes within the PD-1/PD-L1 signaling cascade—such as the phosphatase SHP2—represents an emerging and promising approach to augment the efficacy of cancer immunotherapies.

### 2.4. Targeted inhibition of the PD-1/PD-L1 axis is an effective strategy for cancer treatment

In the TME, PD-L1 expression is significantly upregulated, leading to engagement of the PD-1/PD-L1 axis and subsequent T cell dysfunction. This phenomenon has been documented across multiple cancer types, with distinct regulatory mechanisms and clinical implications. For example, in chronic lymphocytic leukemia (CLL), crosstalk between malignant cells and stromal components drives PD-L1 overexpression via the Notch-c-Myc-EZH2 signaling cascade, thereby enhancing resistance to autologous T lymphocyte-mediated killing 59. In plasmablastic lymphoma (PBL), a subset of cases characterized by elevated PD-L1 expression in both cancer cells and the TME correlates with reduced overall survival 60. In head and neck cancer, aneuploidy-associated chromosome 9p deletion contributes to immune evasion, while arm-level 9p loss accompanied by JAK2-PD-L1 co-deletion serve as a prognostic indicator of poor response to anti-PD-1 immunotherapy 61. Collectively, these findings underscore the critical role of the PD-1/PD-L1 signaling pathway in tumor-mediated immune evasion and highlight the therapeutic potential of targeting this pathway to enhance antitumor immune responses.

Accumulating evidence demonstrates that targeting the PD-1/PD-L1 axis represents a promising strategy for remodeling the TME and enhancing antitumor immunity. In head and neck squamous cell carcinoma (HNSCC), unique spatial distribution characteristics of CD68⁺ and CD163⁺ leukocytes have been identified, with PD-L1-high (PD-L1^hi^) and PD-1-high (PD-1^hi^) cells preferentially accumulating around tumor foci. Notably, elevated peritumoral infiltration densities of PD-L1^hi^CD68^hi^CD163^hi^ cells or PD-1^hi^ T cells correlate with favorable patient survival, suggesting that PD-1/PD-L1 crosstalk between specific cellular subsets within the TME can modulate clinical outcomes 62. In pancreatic ductal adenocarcinoma (PDAC), neoadjuvant radioimmunotherapy combining the GVAX vaccine, anti-PD-1 agents, and stereotactic body radiation (SBRT) reshapes the TME toward an antitumor immune profile. This combinatorial regimen increases infiltration of GZMB⁺CD8⁺ T cells, TH1, and TH17, while concurrently increasing the proportion of immunosuppressive M2-like tumor-associated macrophages (TAMs) 63. In clear cell renal cell carcinoma (ccRCC), engineered chimeric antigen receptor T (CAR-T) cells targeting carbonic anhydrase IX (CAIX) and secreting anti-PD-L1 monoclonal antibodies restore robust antitumor immunity. This effect is achieved by enhancing tumor-killing cytotoxic activity, reducing immunosuppressive cell populations, and strengthening T follicular helper (Tfh)-B cell crosstalk within the TME 64. Collectively, these findings underscore the pleiotropic role of PD-1/PD-L1 interactions in TME remodeling and reinforce that therapeutic targeting of this axis represents a well-established and effective therapeutic strategy for cancer.

## 3. PD-1/PD-L1 inhibitors

### 3.1. PD-L1 inhibitors

Based on their developmental status, PD-L1 small molecule inhibitors can be divided into those that have entered clinical trials and those still in preclinical research. The following section first introduces inhibitors that have reached clinical evaluation, followed by a summary of representative compounds at the preclinical stage.

#### 3.1.1. Clinical-stage PD-L1 inhibitors

Gilead Sciences developed GS-4224 (evixapodlin) (**1**, Table 2), a C2-symmetric tetraaryl PD-L1 inhibitor 65. Its symmetric structure enables simultaneous binding to two PD-L1 molecules, promoting PD-L1 dimerization and thereby blocking the PD-L1/PD-1 interaction (IC_50_ = 0.213 nM). *In vitro*, GS-4224 effectively reversed PD-L1- mediated T cell suppression, augmented immune-mediated tumor cell lysis, and demonstrated activity comparable to the PD-L1 antibody atezolizumab. In MC38 murine models harboring PD-L1 knock-in modification, GS-4224 achieved greater than 90% target occupancy and elicited robust antitumor efficacy. A phase I clinical trial (NCT04049617) reported favorable pharmacokinetics and tolerability, alongside dose-dependent T cell activation and cytokine induction, although the trial has now been terminated 65, 66.

ChemoCentryx Inc. synthesized CCX559 (**2**, Table 2), a substituted biphenyl-based small molecule that promotes PD-L1 dimerization and subsequent internalization, thereby effectively blocking PD-1 engagement (IC_50_ = 0.47 nM) 67. In preclinical murine models, this agent demonstrated significant antitumor activity and induced reversible PD-L1 internalization. A phase I dose-escalation trial (ACTRN12621001342808) confirmed potent inhibition of PD-L1 binding to both PD-1 and CD80 *in vitro*. In MC38 tumor models, cell-surface PD-L1 expression was restored following treatment discontinuation, indicating the reversibility of its mechanism of action 68.

Wu *et al.* reported in a conference presentation that ASC61 (**3**, Table 2) was a PD-L1 inhibitor 69. As a prodrug, this compound was metabolized *in vivo* to form its active metabolite, ASC61A, which induces PD-L1 dimerization and internalization, thereby effectively restoring T cell function. In CT-26-hPD-L1 tumor-bearing BALB/c mouse model, ASC61 exhibited the most potent antitumor activity among all tested compounds evaluated in the study.

MaxiNovel developed MAX-10181 (**4**, Table 2), an active PD-L1 inhibitor that exhibited potent activity and efficacy comparable to that of durvalumab (IC_50_ = 18 nM) 70. A phase I trial (NCT04122339) conducted in Australia and China demonstrated good safety and tolerability, along with consistent pharmacokinetic profiles across diverse ethnic populations. Notably, among subjects with PD-L1 expression levels below 5%, disease control rates were comparable to those achieved with pembrolizumab. Furthermore, meaningful clinical activity responses were observed in individuals who were resistant or intolerant to prior PD-1/PD-L1 antibody therapy 71.

Wang *et al.* synthesized BPI-371153 (**5**, Table 2), a PD-L1 inhibitor that induced PD-L1 dimerization and enhanced its internalization (IC_50_ = 4.2 nM) 72. This compound subsequently activated NFAT signaling and promoted IFN-γ release, leading to restored T cell function. In preclinical models, BPI-371153 significantly inhibited tumor growth and demonstrated favorable pharmacokinetic properties, including high oral bioavailability.

Koblish *et al.* discovered INCB086550 (**6**, Table 2), a biphenyl-based PD-L1 inhibitor 73. In biochemical assays, this compound potently interfered with the PD-1/PD-L1 interaction while simultaneously promoting PD-L1 dimerization and subsequent internalization (IC_50_ = 3.1 nM). Mechanistic studies further revealed that treatment with INCB086550 induced transcriptional programs indicative of T cell activation *in vivo* 73. This finding aligns with clinical observations from a phase I clinical trial (NCT03762447), in which INCB086550 demonstrated dose-dependent T cell activation 74, 75.

Chen *et al.* identified IMMH-010 as a candidate drug after *in vitro* and *in vivo* screening and structural modifications 76, 77. IMMH-010 was identified as a prodrug that undergoes rapid *in vivo* metabolism to yield its bioactive form YPD-29B (**7**, Table 2). YPD-29B potently abrogated the PD-1/PD-L1 interaction (IC_50_ < 0.1 pM) and exhibited a markedly prolonged half-life within tumors relative to plasma, likely due to its high receptor-binding affinity, resulting in sustained intratumoral drug accumulation and improved therapeutic outcomes 78. A phase I trial (NCT04343859) was completed, but its current status is unknown.

#### 3.1.2. Preclinical-stage PD-L1 inhibitors

In addition to the compounds that have reached clinical evaluation, numerous PD-L1 small molecule inhibitors remain at the preclinical stage and display promising *in vitro* and *in vivo* activities.

Hermanowicz *et al.* described MM-129 (**8**, Table 3), a heterofused 1,2,4-triazine derivative 79. This compound inhibited the expression of PD-L1 and induced G_0_/G_1_ phase cell cycle arrest, thereby reducing the proliferative viability of colorectal cancer cells and impairing DNA synthesis (IC_50_ = 3.1 μM in DLD-1 cell) 80. When administered at 10 µmol/kg in murine models, MM-129 significantly impeded tumor progression without inducing nephrotoxicity or adverse hematological effects 81. Furthermore, combination therapy with indoximod (IDO1 inhibitor) enhanced antitumor responses in colon cancer models, suggesting potential for combinatorial immunotherapy approaches 82.

Sun *et al.* designed PD-L1 inhibitors with biphenyl scaffolds, among which S4-1 (**9**, Table 3) exhibited the highest activity 83. This compound interfered with PD-1/PD-L1 binding by inducing PD-L1 dimerization and cellular internalization, leading to its aberrant accumulation in the endoplasmic reticulum and potential proteolytic degradation (IC_50_ = 6.1 nM). *In vitro*, S4-1 markedly boosted T cell activation and strengthened the cytotoxic capacity of peripheral blood mononuclear cells (PBMCs) toward tumor cells. In murine models of lung and colorectal cancer, this compound exhibited robust antitumor efficacy. Notably, in a humanized colorectal cancer model, S4-1 achieved an 88.8% tumor growth inhibition rate, underscoring its promising therapeutic potential.

Prucksaritanont *et al.* developed novel tetra-aryl-scaffold PD-L1 inhibitors using ring fusion design and structural extension strategies, among which CB31 (**10**, Table 3) exhibited the strongest activity in blocking PD-1/PD-L1 binding (IC_50_ = 0.2 nM) 84. This compound triggered the internalization of surface PD-L1, altered its glycosylation pattern leading to intracellular retention, and reduced the total PD-L1 protein levels (IC_50_ = 0.2 nM). It exhibited minimal off-target cytotoxicity, and enhanced TCR-dependent cytokine release as well as PBMC-mediated tumor cell killing.

Wang *et al.* developed APBC (**11**, Table 3), a PD-L1 inhibitor featuring a novel scaffold composed of a biphenyl ring and a carbamoylphenyl group 85. This compound directly bound to the PD-L1 dimer, locked its conformation, and blocked the PD-1 binding site (IC_50_ = 27.82 μM). In MC38 mouse tumor models, APBC significantly increased the infiltration density of CD4⁺ and CD8⁺ T cells in tumor tissue, promoted cytokines production without inducing hepatotoxicity, and exhibited superior therapeutic efficacy and plasma stability.

Mao *et al.* developed Pt-2 (**12**, Table 3), a cyclometalated platinum-metformin conjugate that functions as a small molecule PD-L1 inhibitor 86. The cyclometalated platinum moiety played multiple pivotal roles: it markedly enhanced the intracellular delivery efficiency of metformin, enabled its selective accumulation in lysosomes, endowed the conjugate with favorable photophysical properties for real-time cellular imaging, and provided potent chemotherapeutic activity against tumor cells. Notably, Pt-2 selectively accumulated in lysosomes, where it not only disrupts the PD-1/PD-L1 signaling pathway at the cell surface but also inhibited PD-L1 expression by the AMPK-TFEB pathway. Specifically, it activated AMPK to promote TFEB-mediated lysosomal biogenesis and function, thereby facilitating the lysosomal degradation of PD-L1 while concurrently suppressing its expression (IC_50_ = 490 nM).

Zhang *et al.* characterized SA-49 (**13**, Table 3) as a PD-L1 inhibitor within a library of novel aloperine analogs 87. At the cellular level, SA-49 reduced both constitutive and IFN-γ-induced PD-L1 expression in NSCLC cells in a time- and concentration-dependent manner, exerted extremely low cytotoxicity on NSCLC cells, and enhanced the cytotoxicity of co-cultured T cells and NK cells against tumor cells. *In vivo*, SA-49 significantly inhibited the growth of Lewis tumor xenografts in C57BL/6 mice via intragastric administration, decreased the expression of PD-L1 in tumor tissues, increased the number of CD3^+^ T cells and reduced the number of FoxP3^+^ Treg cells, thus activating the TME. Meanwhile, it had no obvious effects on the body weight, major organs and serum biochemical indicators of mice, with no significant systemic toxicity observed.

Bristol-myers squibb (BMS) developed BMS-202 (**14**, Table 3) as a lead prototypical compound via structural optimization aligned with the hydrophobic pocket characteristics of the PD-L1 dimerization interface, complemented by comprehensive verification of its biological activity and underlying molecular mechanism 88-90. BMS-202 disrupted PD-1/PD-L1 receptor-ligand complex formation by binding to a hydrophobic cleft generated upon PD-L1 dimerization (IC_50_ = 18 nM) 26. Beyond immune checkpoint blockade, BMS-202 modulated extracellular matrix biology by suppressing type I collagen synthesis in fibroblasts and promoting branched-chain amino acid transaminase 1 (BCAT1) expression, thereby enhancing L-isoleucine catabolism and impairing the malignancy of glioblastoma multiforme. BMS-1166, an optimized derivative of BMS-202, introduced a 1,4-benzodioxane moiety at the terminus of the biphenyl ring and incorporated an m-cyanobenzyl alcohol group into the central benzene ring 91, 92. This derivative induced PD-L1 dimerization, a mechanism consistent with that of BMS-202. However, BMS-1166 exhibited a significantly enhanced binding affinity, with its IC_50_ value decreased drastically to 1.4 nM.

**Figure 4:**
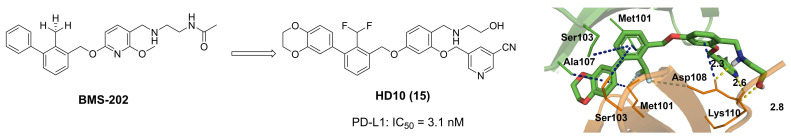
Discovery of HD10 and its co-crystal structure with PD-L1 (PDB ID: 9ERY). Yellow dashed lines depict hydrogen bonds. Gray dashed line indicates an electrostatic interaction. Blue dashed line highlights a π-sulfur interaction.

Zhang *et al.* designed and developed compound HD10 (**15**, Table 3) as a novel PD-L1 inhibitor (IC_50_ = 3.1 nM) based on BMS-202 (Figure 4) 93. HD10 bound PD-L1 with high affinity, and its co-crystal structure revealed critical interactions between its difluoromethylbiphenyl core and the PD-L1 dimer interface (PDB ID: 9ERY). At the cellular level, HD10 could effectively block the binding between hPD-1 293T cells and hPD-L1, promote the secretion of IFN-γ in PBMCs in a dose-dependent manner to restore T cell function without significant toxicity to PBMCs. It could significantly induce apoptosis in HCC827 and MDA-MB-231 cells with high PD-L1 expression, but had no obvious effect on A549 cells with low PD-L1 expression. *In vivo*, when administered orally at 50 mg/kg in the PD-1/PD-L1 humanized mouse model, the tumor growth inhibition rate (TGI) reached 57.31% without obvious toxicity. It could activate the immune system by increasing the proportion of tumor infiltrating CD3⁺ and CD8⁺ T cells, upregulating the expression of CXCR3/CXCL9/CXCL10 chemokines, and enhancing the levels of granzyme B and perforin. In addition, HD10 possesses favorable pharmacokinetic properties.

Wang *et al.* designed oxadiazole-based structures, among which compound 16 (Table 3) exhibited the strongest ability 94. This compound bound to both human and murine PD-L1 and induced its dimerization (IC_50_ = 27.8 nM). It promoted time- and concentration-dependent internalization of cell-surface PD-L1, followed by degradation via a lysosome-dependent pathway, while upregulating the expression of lysosome-related genes. *In vitro*, it attenuated PD-1/PD-L1 binding and activated PBMCs-mediated antitumor immunity in a dose-dependent manner. *In vivo*, oral administration exhibited dose-dependent antitumor effects in both CT26 colon cancer and B16-F10 melanoma mouse models. At a dose of 160 mg/kg, it achieves a 75% tumor growth inhibition rate of CT26 tumors without obvious toxicity. Mechanistically, high-doses treatment reduced PD-L1 expression in tumor tissues, increased CD8⁺ cell infiltration, and elevated the expression of immune factors.

Xiao *et al.* designed PD-L1 inhibitors by introducing hydrophilic tail groups at the end of the biphenyl skeleton, among which NPH 16 (**17**, Table 3) exhibited the most potent activity (IC_50_ = 24.4 nM) 95. It bound efficiently to the PD-L1 dimer through hydrogen bonds and hydrophobic interactions, and showed concentration-dependent binding ability to both human and murine PD-L1. In the HepG2/Jurkat coculture model, NPH 16 promoted tumor cell apoptosis in a dose-dependent manner. *In vivo*, it increased the infiltration of CD3⁺CD8⁺T cells and the level of IFN-γ in tumor tissues, while reduced PD-L1 expression, with no obvious organ toxicity. Meanwhile, it possessed excellent drug-like properties with a water solubility of 0.794 mg/mL and an oral bioavailability of 15.9%.

Lu *et al.* synthesized D6 (**18**, Table 3), a derivative based on the 5-phenylindoline 96. This compound formed a stable binding with the hydrophobic pocket of the PD-L1 dimer, thereby blocking the PD-L1/PD-1 interaction (IC_50_ = 4.8 nM). *In vitro*, 100 nM D6 significantly promoted IFN-γ secretion from PBMCs and inhibited immune cells apoptosis. *In vivo*, D6 exhibited dose-dependent tumor growth inhibitory in the MC38 tumor-bearing mouse models. Mechanistically, it activated the TME by enhancing the infiltration and activation of CD8⁺ T cells, upregulating the levels of cytokines such as IFN-γ, and inhibiting tumor cell proliferation.

Moore *et al.* identified ARB-272572 (**19**, Table 3) as a potent PD-L1 inhibitor through a small molecule library 97. Subsequent research demonstrated that ARB-272572 triggered PD-L1 dimerization and subsequent internalization into the cytosol, leading to reduced cell surface PD-L1 levels (IC_50_ = 400 pM). In a humanized PD-1/PD-L1 colorectal cancer mouse model, administration at 10 mg/kg for 7 days reduced average tumor volume by 60.4%, accompanied by decreased PD-L1 expression in tumors, increased peripheral blood CD3^+^/CD4^+^ T cells, and a reduction in regulatory T cells. In patients with chronic hepatitis B, ARB-272572 decreased the PD-L1 expression on the surface of dendritic cells and B cells, significantly enhanced the proliferation of HBV-specific T cells and IFNγ secretion, and elevated the frequency of HBsAg-specific B cells. These findings highlight the dual biological activity of ARB-272572 against both tumors and chronic viral infections.

Feng *et al.* conducted a virtual screening of 3,906 small molecules and approved drugs, including those used for immune regulation and antitumor traditional Chinese medicine, and identified anidulafungin (**20**, Table 3) inhibited PD-L1 expression 98. Research found that anidulafungin directly bound to PD-L1, decreasing the PD-1/PD-L1 intermolecular binding energy from -63.9 kcal/mol to -36.8 kcal/mol, forcing PD-1 to deviate from its original binding site. In addition, it activated both systemic and local antitumor immunity, demonstrating markable antitumor efficacy in both *in vitro* and *in vivo* experimental models.

Wang *et al.* identified CBPA (**21**, Table 3) as a potent inhibitor of PD-1/PD-L1 signaling through screening 99. CBPA bound to dimeric PD-L1 and occluded the PD-1 interaction surface on PD-L1. It restored T cell function and significantly promoted the secretion of proinflammatory cytokines such as IFN-γ and TNF-α by primary CD4^+^ T cells. *In vivo,* in the MC38 colon adenocarcinoma and B16F10 melanoma mouse models, intraperitoneal injection of CBPA at 10 mg/kg achieved tumor growth inhibition rates of 67.20% and 45.26% respectively, without obvious liver or kidney function damage or body weight loss. Mechanistically, CBPA significantly increased the infiltration of CD4^+^ and CD8^+^ T cells in the TME, promoted the secretion of perforin and granzyme B by intratumoral CD8^+^ T cells, and upregulated immune-related pathways such as antigen processing and presentation and T cell receptor signaling.

In the field of PD-1/PD-L1-targeted small molecule cancer immunotherapy, biphenyl-based and C2-symmetric tetraaryl compounds represent the most clinically translatable core chemotypes. These agents exert their effects either by inducing PD-L1 dimerization and internalization or by directly blocking the protein-protein interactions. Several such molecules have entered phase I clinical trials, demonstrating favorable pharmacokinetic profiles and promising antitumor activity. However, the field still faces multiple challenges associated with clinical failure, including pharmacokinetic issues (such as short half-lives and poor water solubility) 100, insufficient selectivity leading to off-target toxicity 101, immune-related adverse events (irAEs) 102, *in vitro* assay artifacts 103, clinical translation biases caused by species differences 104, and inadequate verification of target engagement 105. When evaluating the strength of evidence in drug development, human target engagement date and clinical outcomes represent the most reliable validation, as their directly determine the clinical utility of a candidate drugs; *In vivo* studies serve as a critical intermediate link by recapitulating the complexity of the TME, thereby enabling assessment of antitumor activity, safety, and pharmacokinetic/pharmacodynamic (PK/PD) relationships; while cell assays are core screening tools for drug discovery, enabling rapid validation of targeted activity, preliminary safety, and structure-activity relationships (SAR), though they are limited by the gap between *in vitro* environments and the actual TME. Collectively, these three types of evidence form a complete chain for drug development, with their respective strengths and limitations complementing each other to support the advancement of PD-1/PD-L1-targeted small molecule therapeutics.

### 3.2. PD-1 inhibitors

In contrast to PD-L1 inhibitors, the development of small molecules targeting PD-1 itself has progressed more slowly. All currently reported compounds remain at the preclinical stage, and none has yet entered clinical trials.

**Figure 5:**
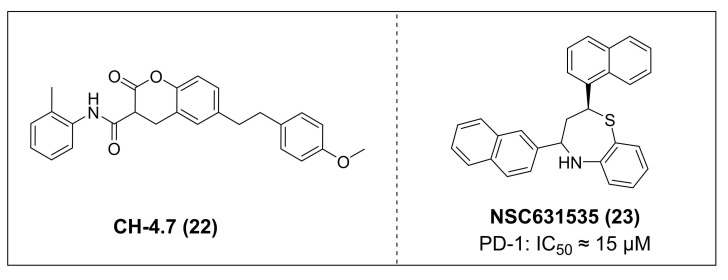
Structures of PD-1 inhibitors.

Lu *et al.* discovered agents with potent inhibitory activity against the PD-1/PD-L1 interaction 106. Among these, CH-4 exhibited the most potent ability to disrupt the binding between soluble PD-L1 (sPD-L1) and membrane-localized PD-1 in KG-1 cells. Subsequent structural optimization led to the identification of CH-4.7 (**22**, Figure 5), an analog of CH-4 that retains strong targeting ability while exhibiting minimal cytotoxicity. Specifically, CH-4.7 exhibited no cytotoxicity against Jurkat cells and KG-1 cells. It effectively blocked the binding of sPD-L1 to PD-1 on the surface of KG-1 cells without altering PD-1 protein expression, and its PD-1/PD-L1 inhibitory activity remains comparable to that of CH-4. Meanwhile, CH-4.7 reversed the inhibition of sPD-L1 on T cells cytokine secretion, significantly elevated effect of IL-2 and IFN-γ levels in PMA/PHA-activated Jurkat cells, maintained T cells activation function. Notably, its inhibitory efficacy at the cellular level surpasses that of PD-1 antibodies.

Patil *et al.* identified NSC631535 (IC_50_ ≈ 15 μM) (**23**, Figure 5) as a PD-1/PD-L1 inhibitor through screening of lead-like and large molecule databases 107. Mechanistically, NSC631535 bound directly to PD-1, thereby inhibiting the PD-1/PD-L1 interaction. *In vitro*, at a concentration of 50 μM, NSC631535 exhibited an inhibitory rate of up to 65.5%. Dose-dependent experiments further revealed that its inhibitory activity is concentration-dependent.

Investigations into small molecule agents targeting the PD-1/PD-L1 axis have revealed that the research focus on PD-L1-targeted strategies 108. These strategies include inhibitors that bind to the hydrophobic pocket of PD-L1 and induce its dimerization 109. This research preference is largely attributable to the unique structural and biological properties of PD-L1. Structurally, its extracellular domain possesses well-defined dimerization interfaces and rigid hydrophobic pockets 109. Biologically, PD-L1 expression is largely restricted to tumor tissues, making its blockade more likely to result in localized immune reactivation in the TME while minimizing systemic immune toxicity 110. The core challenges in this area lie in the balance of efficacy and the optimization of druggability 111. In contrast, PD-1 presents substantially greater challenges as a small-molecule target. Its extracellular binding region exhibits conformational flexibility and lacks stable hydrophobic pockets, rendering it less druggable 112, 113. Moreover, as a core immune checkpoint receptor, PD-1 is expressed on various immune cells types and plays a critical role in maintaining systemic immune tolerance 114. Blockade of PD-1 therefore leads to widespread immune activation, increasing the risk of irAEs. In addition, PD-1 can bind to PD-L1 and PD-L2. Selective inhibition of PD-L1 preserves the PD-1/PD-L2 axis, thereby helping to maintain immune homeostasis. Conversely, PD-1 inhibition disrupts both pathways, potentially leading to immune overactivation and an elevated risk of autoimmune reactions 115, 116. These mechanistic and pharmacological distinctions have shaped the current landscape of drug development for the PD-1/PD-L1 pathway. Currently, antibody-based therapies dominate PD-1-targeted strategies, while small molecule drug development faces bottlenecks such as low binding affinity and insufficient selectivity 117. As a result, PD-L1 has emerged as the mainstream target for small-molecule inhibitor development, while PD-1-targeted therapies remain primarily antibody-based, with small-molecule candidates lagging considerably behind in clinical progress 118.

## 4. PD-L1 degraders

### 4.1. PD-L1 PROTACs

PROTACs are heterobifunctional molecules that harness the ubiquitin-proteasome pathway to induce selective degradation of target proteins. These molecules consist of three key structural components: a ligand that interacts with target protein, an E3 ubiquitin ligase-recruiting ligand, and a chemical linker that connects these two functional moieties 119. PROTACs exert their activity by promoting ubiquitination modification of the target protein, thereby flagging it for proteasomal recognition and degradation. This unique strategy not only enables effective modulate disease-associated proteins but also extends therapeutic intervention to protein subtypes traditionally considered “undruggable” or those that have developed resistance to conventional inhibitors. As such, PROTAC technology has opened new research avenues and expanded the therapeutic landscape in immuno-oncology and beyond 120, 121.

**Figure 6:**
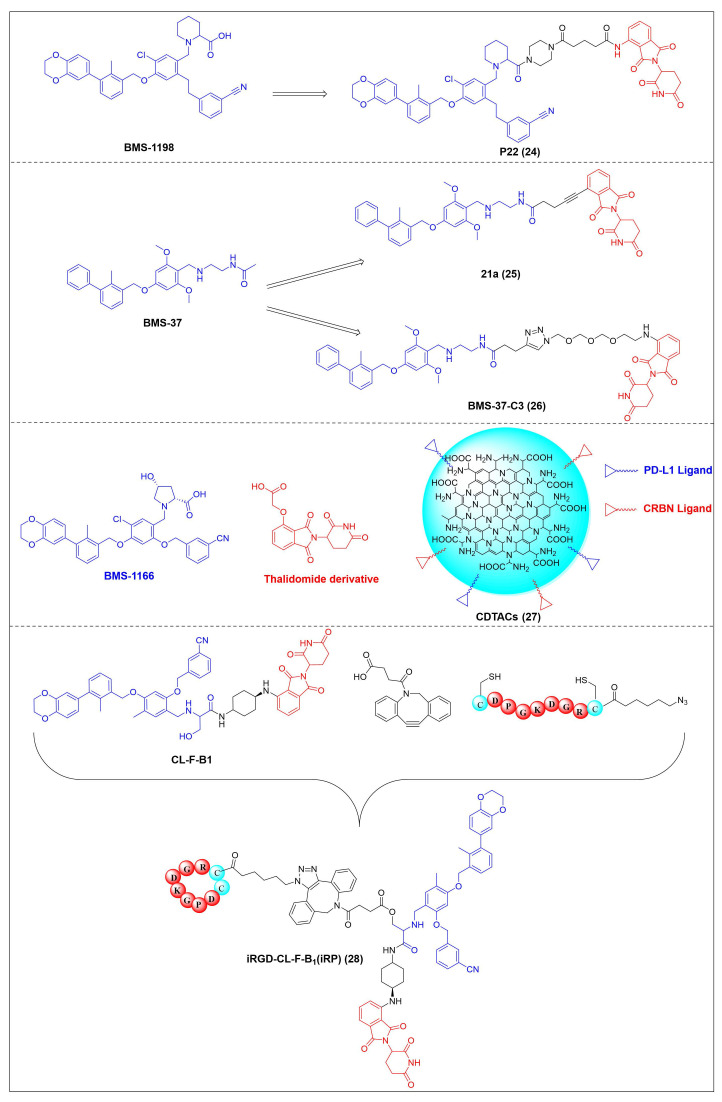
Structures of PD-L1 PROTACs.

Chen *et al.* developed compound P22 (**24**, Figure 6) by conjugating the PD-L1 inhibitor BMS-1198 with the E3 ligase ligand pomalidomide via a rigid piperazine linker 122. Compared to flexible or straight linkers, this rigid linker was more conducive to preserving the binding affinity between the molecule and PD-L1. In a Hep3B/OS-8/hPD-L1 and CD3^+^ T cell coculture model, P22 significantly promoted IFN-γ secretion in a dose-dependent manner, exhibiting efficacy superior to that of the clinical drug Keytruda. Furthermore, P22 moderately reduced PD-L1 protein level in MDA-MB-231 cells, and this degradation effect was dependent on the lysosomal pathway rather than the proteasomal pathway. For membrane proteins like PD-L1, PROTACs could induce protein endocytosis followed by lysosomal degradation. This phenomenon was a common pathway for PROTAC-mediated membrane protein degradation and not exclusive to LYTACs 123.

Wang *et al.* conjugated BMS-37 with thalidomide to generate a series of PROTACs, from which compound 21a (**25**, Figure 6) was identified as the most promising PD-L1 degrader 124. *In vitro*, 21a degraded PD-L1 in a dose- and time-dependent manner via a proteasome-dependent pathway in various malignant cell lines including hematological, breast, colorectal and prostate cancer cell. Notably, it preferentially degraded cytoplasmic PD-L1 for degradation, thereby reducing its expression on the cell membrane. *In vivo*, 21a significantly decreased PD-L1 levels in MC-38 tumors, promoted CD8^+^ T cell infiltration, and upregulated genes associated with CD8^+^ T cell cytotoxicity. These effects translated into potent tumor growth inhibition without causing changes in mouse body weight.

Liu *et al.* synthesized PD-L1 degraders by conjugating BMS-37 with thalidomide via PEG-based linkers 125. Among these, BMS-37-C3 (**26**, Figure 6) emerged as the most potent degrader. In A375 and B16-F10 melanoma cells, BMS-37-C3 degraded PD-L1 in a dose- and time-dependent manner. In the coculture model comprising A375 cells and T cells, BMS-37-C3 enhanced T cell-mediated tumor killing in a concentration-dependent fashion. Notably, its antitumor efficacy surpassed that of both the parent compound BMS-37 and the marketed monoclonal antibody atezolizumab.

Su *et al.* developed a class of carbon dot-based PROTACs (CDTACs) (**27**, Figure 6) by conjugating the PD-L1-binding probe BMS-1166 and the CRBN E3 ligase ligand thalidomide onto carbon dots (CDs) 126. CDs are biocompatible, photostable, and fluorescently traceable nanomaterials that enable tumor accumulation via both the enhanced permeability and retention (EPR) effect and active PD-L1 targeting. In 2-12 h following intravenous injection, CDTACs accumulated in tumors through EPR and active targeting, with accumulation further enhanced by folic acid-modified dextran (FMD). Treatment with CDTACs combined with FMD increased intratumoral infiltration of CD8⁺ T cells, elevated levels of pro-inflammatory cytokines such as IFN-γ and TNF-α, and reduced immunosuppressive factors including IL-4 and IL-10. Notably, CDTACs efficiently degraded PD-L1, reshaped the TME, and demonstrated potent antitumor efficacy even in PD-L1 inhibitor-insensitive tumors such as B16-F10 melanoma.

Qi *et al.* connected BMS-1001 with pomalidomide through a rigid *trans*-1,4-diaminocyclohexyl linker to obtain CL-F-B_1_ 127. In MC38 colon cancer cells, CL-F-B_1_ achieved 67.05% PD-L1 degradation at 5 μM after 24 h of incubation, with a DC_50_ of 2.32 μM. To address the challenge of targeted delivery, they further conjugated CL-F-B_1_ with the cyclic iRGD peptide via azide-alkyne cycloaddition (click chemistry) to generate the iRGD-CL-F-B₁ conjugate (iRP) (**28**, Figure 6). This conjugate could self-assemble into nanomicelles (iRP NPs), the iRGD peptide could mediate deep tumor penetration via αvβ3 integrins and neuropilin-1 (NRP-1). *In vitro*, iRP NPs exhibited 3.78-fold higher cellular uptake in MC38 cells efficiency compared to control PROTAC NPs. *In vivo*, iRP NPs exhibited high intratumoral accumulation in the MC38 tumor model. At a dose of 6 mg/kg, the formulation achieved an 80.88% TGI, promoted CD8^+^ T cell infiltration, and caused no damage to major organs.

### 4.2. PD-L1 LYTACs

LYTACs encompass three key structural components: a target protein-binding ligand, a ligand specific for the lysosomal targeting receptor (LTR), and a connecting linker 128. Following binding to their respective targets, LYTACs assemble a ternary molecular complex with the LTR, which subsequently undergoes receptor-mediated endocytosis and lysosome-dependent degradation.

**Figure 7:**
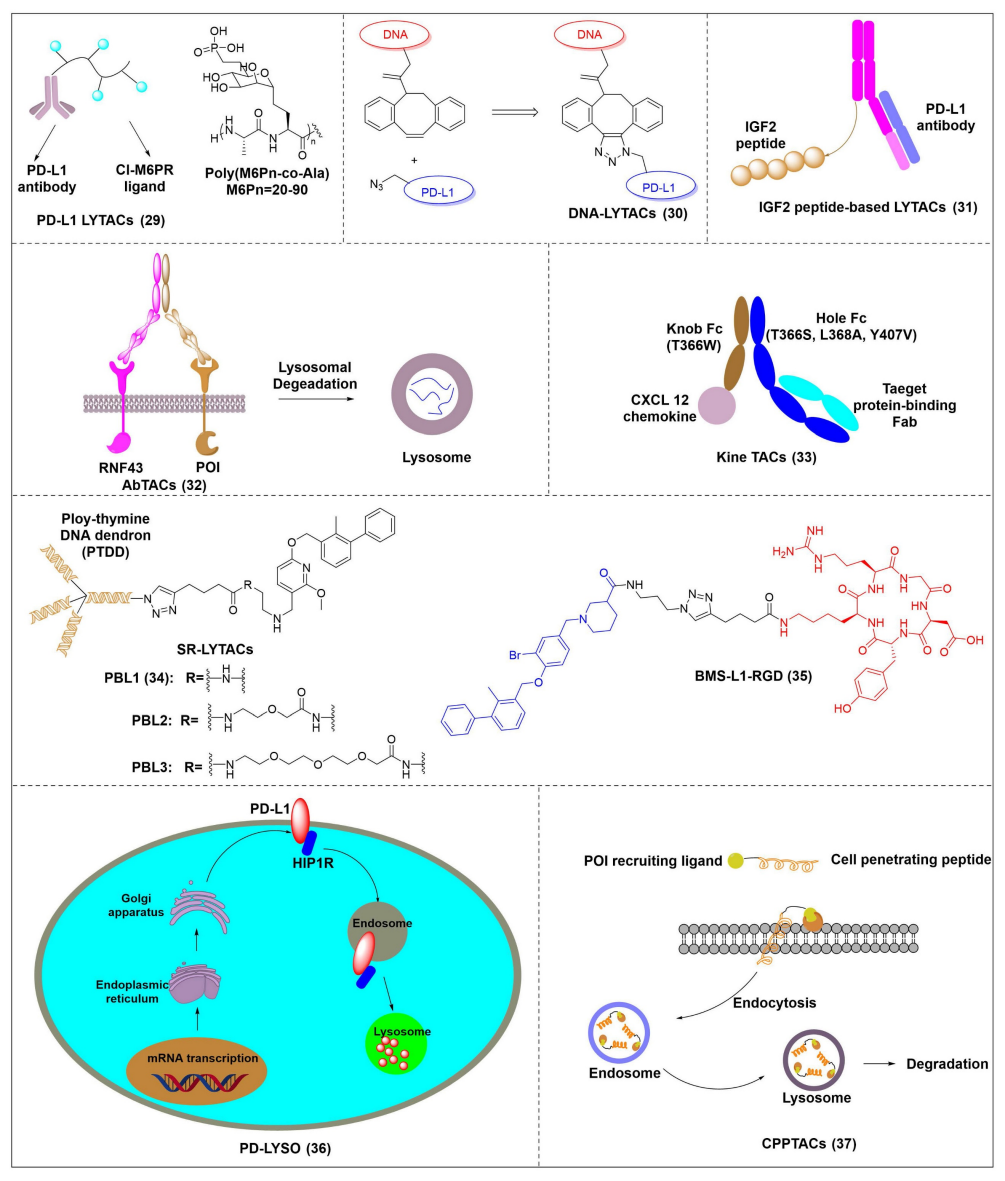
Structures of PD-L1 LYTACs.

Banik *et al.* utilized the cation-independent mannose-6-phosphate receptor as LTRs by conjugating the PD-L1 antibody atezolizumab with a glycopeptide ligand that targets the cation-independent mannose-6-phosphate receptor (CI-M6PR), which resulted in the production of atz-LYTAC (**29**, Figure 7) 129. The degradation activity of atz-LYTAC was associated with the cell surface expression level of CI-M6PR. In MDA-MB-231 cells, which exhibit low CI-M6PR expression, atz-LYTAC could reduce the cell surface PD-L1 levels by approximately 33%. In contrast, in HDLM-2 cells with high CI-M6PR expression, treatment with atz-LYTAC achieved approximately 70% PD-L1 degradation within 48 hours, which was significantly superior to that of the unconjugated parental antibody.

Liu *et al.* developed DNA aptamer-based covalent LYTACs (**30**, Figure 7) that targeting the CI-M6PR on one side and enabling bioorthogonal covalent conjugation-facilitated specific binding to PD-L1 on the other 130. These covalent LYTAC not only abrogated the immune suppression of the PD-1/PD-L1 axis but also was the first found to inhibit STAT3 phosphorylation by degrading PD-L1, directly inducing immunogenic apoptosis of tumor cells, releasing damage-associated molecular patterns such as calreticulin, and promoting dendritic cell maturation and tumor-specific cytotoxic T cell responses.

Sitar *et al.* developed IGF2- derived peptides as ligands for CI-M6PR, which were subsequently fused to a PD-L1 antibody to generate peptide/protein-based LYTACs (**31**, Figure 7) 131. The designed IGF2-derived polypeptides bound to IGF2R with high affinity and specificity and did not bind to IGF1R. Their corresponding LYTACs exhibited bispecific binding to both PD-L1 and IGF2R, induced the internalization and degradation of soluble and transmembrane PD-L1 in a time- and concentration-dependent manner, and significantly triggered the growth inhibition and lysis of tumor cells when co-incubated with PBMCs, with efficacy superior to that of traditional anti-PD-L1 antibodies. Meanwhile, this class of LYTACs showed no obvious cytotoxicity on their own.

Cotton *et al.* developed a novel cell-surface protein degradation technology called antibody-based PROTACs (AbTACs) (**32**, Figure 7) 132. AbTACs induced internalization and lysosomal degradation of target proteins by connecting cell surface proteins with E3 ubiquitin ligase RNF43. The team developed a bispecific antibody named AC-1. Incubation of cells with 10 nM AC-1 for 12 hour initiated PD-L1 degradation, and maximal effect was achieved at 24 hours (D_max_ = 63%, DC_50_ = 3.4 nM). In terms of broad-spectrum activity, treatment with 10 nM AC-1 for 24 hours achieved PD-L1 degradation in PD-L1-high-expressing cell lines of triple-negative breast cancer, non-small cell lung cancer and bladder cancer.

Wells *et al.* developed cytokine receptor-targeting chimeras (KineTACs) that exploits endogenous cytokine-mediated internalization of cognate receptors to deliver target proteins for lysosomal degradation 133. Using a CXCL12-based KineTAC conjugated to a PD-L1 antibody, they successfully induced PD-L1 degradation in MDA-MB-231 cells. The degradation efficiency of this KineTAC correlated strongly with the binding affinity and dissociation rate of the antibody arm for PD-L1, while remaining unaffected by CXCR4 signaling or pH-dependent binding. The system exhibited high selectivity, downregulating PD-L1 without affecting other surface proteins, and retained its activity against murine PD-L1 in MC38 and CT26 cells.

Huang *et al.* reported the first DNA-based lysosome-targeted degradation strategy mediated by scavenger receptors (SRs), establishing SRs as novel LYTACs receptors 134. By coupling polythymidine DNA dendrites with BMS-202, three compounds with different linker lengths (PBL1, PBL2, and PBL3) were synthesized. Among these, PBL1 (**34**, Figure 7) exhibited the most potent activity (DC_50_ = 110.4 nM) and effectively promoted PD-L1 degradation in cancer cells. Mechanistically, PBL1 was internalized by cells and targeted to lysosomes in a concentration- and time-dependent manner. In the immune co-culture model combined with PBMCs, PBL1 effectively enhanced the killing effect of immune cells on tumor cells. Collectively, PBL1 exhibited potent PD-L1 degradation activity, low off-target toxicity, and excellent anti tumor efficacy both *in vitro* and *in vivo*.

Zheng *et al.* designed cyclic Arg-Gly-Asp (cRGD)—a ligand for integrin αVβ3—and conjugated it to the BMS-8 via various linkers, generating three integrin-facilitated lysosomal degraders (IFLDs) for PD-L1 targeting 135. Under the action of IFLDs, the target protein formed a ternary complex with the IFLD molecules and integrins, which was sequentially accompanied by endocytosis and lysosomal degradation. Among the constructs, BMS-L1-RGD (**35**, Figure 7) exhibited the most potent PD-L1 degradation activity in MDA-MB-231 cells. In the B16F10 tumor xenograft mouse model, BMS-L1-RGD significantly inhibited tumor growth and reduced tumor weight, markedly downregulated PD-L1 expression in tumor tissues and significantly elevated the level of tumor cell apoptosis. Furthermore, it decreased splenic metastasis, with no obvious body weight loss or other side effects were observed in the mice.

Xu *et al.* identified huntingtin-interacting protein 1-related (HIP1R) as an endogenous ligand that bound to the intracellular domain of PD-L1 and mediated its degradation via a lysosome-dependent pathway 136. Based on this mechanism, the authors designed a chimeric peptide, designated PD-LYSO (**36**, Figure 7), which effectively induces lysosome-dependent degradation of PD-L1. By decreasing PD-L1 expression, PD-LYSO reduced the binding of tumor cells to PD-1, thereby enhancing T cell-mediated cytotoxicity.

He *et al.* developed a platform of CPP-mediated lysosome-targeting chimeras by exploiting the endosomal entrapment effect of cell-penetrating peptides (CPPs) and conjugating different CPPs to target small protein-binding molecules 137. By linking BMS-8 with the CPP penetrating through a triazole linker, the authors generated a BMS-CPP conjugate (**37**, Figure 7). This conjugate could significantly degrade PD-L1 via lysosomal pathways (up to 75%-80%) without the “hook effect”, a common limitation of certain degradation platforms. Notably, BMS-CPP offered broad applicability—it was applicable to almost all cell types, exhibited high linker tolerance, possessed good tissue penetration, and had no immunogenicity.

This chapter focuses on small molecule PD-L1 degraders and provides a systematic overview of two core technologies: PROTACs and LYTACs. The former degrades PD-L1 via the ubiquitin-proteasome pathway, while the latter accomplishes this through receptor-dependent endocytosis and lysosomal degradation. Both types of degraders have developed innovative delivery systems such as carbon dots, iRGD-modified nanomicelles, and CPPs to enhance tumor targeting and tissue penetration. They have demonstrated significant tumor-suppressive effects in preclinical models and can exert efficacy against PD-L1 inhibitor-insensitive tumors. However, current research still has obvious limitations. First, all reported degraders remain in the preclinical stage, lacking support from human clinical trial data, and species differences may affect translational outcomes 138, 139. Second, technical bottlenecks such as the difficulty in large-scale production of delivery systems, the impact of PROTACs linker design on degradation efficiency, and the immunogenicity risk of LYTACs have not been fully overcome 129, 140, 141. Third, the structure-activity relationships (SAR) underlying degradation mechanisms remain insufficiently characterized 142. Addressing these challenges will be critical for advancing PD-L1 degraders toward clinical application.

## 5. Co-inhibitors of PD-L1 and other targets

The clinical applicability of PD-1/PD-L1 monotherapies is often constrained by limited response rates, selectivity challenges, and acquired resistance 143. Dual-target inhibitors offer a strategy to mitigate these limitations by exerting synergistic effects, enabling more durable and comprehensive tumor control, and potentially lowering toxicity compared to combination regimens. This approach represents a promising avenue for precision immuno-oncology 144. Herein, we summarize the reported dual-targeted agents that combine PD-L1 inhibition with the modulation of key secondary targets, highlighting their therapeutic rationale and potential.

### 5.1. PD-L1/ HDAC inhibitors

Histone deacetylases (HDACs) are enzymes that catalyze the deacetylation of lysine residues on proteins. By modulating chromatin structure and non-histone protein functions, HDACs negatively regulate gene transcription at the epigenetic level and play a crucial role in cell proliferation, differentiation, apoptosis, and immune regulation 145. HDAC can upregulate the expression of PD-L1 through epigenetic regulation and signaling pathway interactions in the TME promoting immune escape 146.

**Figure 8:**
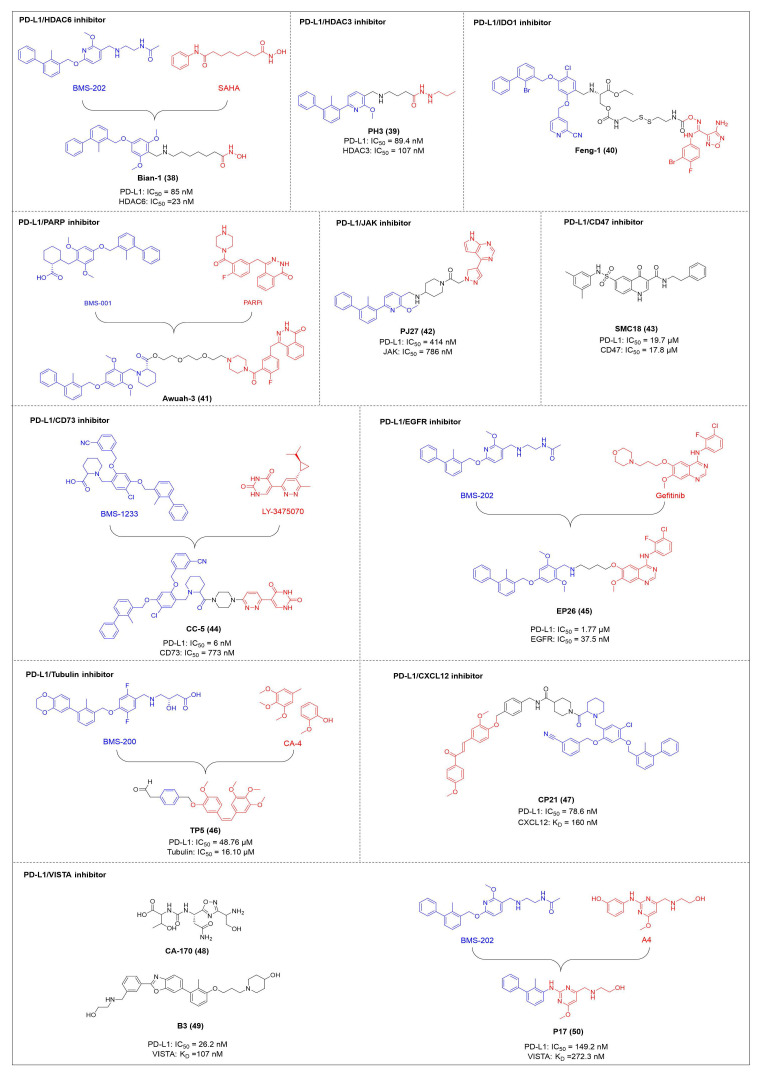
Structures of co-inhibitors of PD-L1 and other targets.

HDAC6 is a multifunctional cytoplasmic deacetylase involved in diverse pathological processes, including cancer and neurodegenerative diseases, through its regulation of microtubule stability and clearance of misfolded protein aggregates 147. Notably, HDAC6 facilitated tumor immune evasion by deacetylating and stabilizing PD-L1 in tumor cells, thereby positioning it as a key therapeutic target that bridges epigenetic regulation and cancer immunotherapy 148. Therefore, inhibiting HDAC6 exhibits a synergistic effect with anti-PD-L1 therapy 149. Bian *et al.* synthesized a PD-L1/HDAC6 dual inhibitor, Bian-10 (PD-1/PD-L1: IC_50_ = 85 nM, HDAC6: IC_50_ = 23 nM) (**38**, Figure 8; Table 4) 150. *In vitro*, it showed significant inhibitory activity on the proliferation of tumor cells such as B16 and CT26. *In vivo*, for the CT26 colon cancer cell xenograft model in nude mice, the tumor inhibition rates of the 50 mg/kg and 100 mg/kg dose groups reached 55% and 75%, respectively, which were superior to those of the positive control drugs BMS-202 (51%) and SAHA (53%).

HDAC3, a class I HDAC family member, regulates the acetylation levels of histone and non-histone proteins, thereby influencing tumor progression and representing an important antitumor target 151. Notably, combining HDAC3 inhibitors with PD-L1 antibodies has been shown to produce synergistic antitumor effects 152. Wang *et al.* synthesized dual-target candidate drugs PH3 (**39**, Figure 8; Table 4) by incorporating the pharmacophores of HDAC3 inhibitors into the tails of PD-L1 inhibitors 153. PH3 exerted potent inhibitory effects on both PD-1/PD-L1 (IC_50_ = 89.4 nM) and HDAC3 (IC_50_ = 107 nM), with remarkable selectivity over other HDAC isoforms. *In vitro*, PH3 inhibited the proliferation of various tumor cells, induced tumor cell apoptosis, and arrested the cell cycle at the G_0_/G_1_ phase. Additionally, in the coculture model, PH3 enhanced T cell-mediated tumor cell killing. *In vivo,* PH3 exhibited a dose-dependent antitumor effect in the B16-F10 melanoma mouse model, which were superior to those of NP19 monotherapy and the combination therapy of NP19+MS-275. No obvious toxicity was observed. Meanwhile, it increased the infiltration of CD3⁺CD8⁺ and CD3⁺CD4⁺ cells in the TME and activated antitumor immunity.

### 5.2. PD-L1/IDO1 inhibitors

Indoleamine 2,3-dioxygenase 1 (IDO1) catalyzes the depletion of tryptophan and the generation of kynurenine, thereby suppressing T-cell functional and promoting immune tolerance 154. The concurrent PD-L1 and IDO1 inhibition can potentiate antitumor immunity 155. Feng *et al.* designed PD-L1/IDO1 dual-target inhibitors incorporating a disulfide bonds linker. This disulfide bonds linker can only be cleaved in the TME/cells, enabling the targeted release of PD-L1/IDO1 inhibitors and thereby reducing off-target toxicity 156. *In vivo*, Feng-1 (**40**, Figure 8; Table 4) exhibited significant antitumor activity in the mouse melanoma B16F10 subcutaneous xenograft model. In the mouse colon cancer CT26 model, Feng-1 achieved a TGI of 54.9%, outperforming both the PD-L1 inhibitor monotherapy (TGI = 40.6%) and the IDO1 inhibitor monotherapy (TGI = 32.1%). Regarding safety, Feng-1 treatment did not significantly affect body weight, spleen weight, or spleen index in mice, and induced a decreasing trend in regulatory T cell (CD4⁺CD25⁺Foxp3⁺) in the spleen.

### 5.3. PD-L1/PARP inhibitors

Poly (ADP-ribose) polymerases (PARPs) are a family of enzymes involved in DNA damage recognition and repair. They maintain genomic stability by catalyzing ADP-ribosylation (PARylation), thereby regulating the function of diverse proteins 157. Research has shown that PARP inhibition can upregulate PD-L1 expression 158, and combined inhibition of PD-L1 and PARP has demonstrated synergistic antitumor effects in preclinical models 159. Ofori *et al.* developed conjugates by linking BMS-001 to a PARP inhibitor pharmacophore 160. Among them, Awuah-3 (**41**, Figure 8; Table 4) retained structural integrity upon incubation in PBS, DMEM medium and human liver microsomes. It exerted potent cytotoxicity across multiple cancer cell lines, including ovarian, lung and breast cancer cells, with activity 2- to 20-fold higher than that of the individual parent drugs. Notably, Awuah-3 showed exceptionally strong killing activity against the triple-negative breast cancer cell line MDA-MB-231, inducing early and late apoptosis in 60% of treated cells. In addition, Awuah-3 significantly downregulated the cell surface PD-L1 expression with an inhibitory effect superior to that of BMS-001. It also arrested the cell cycle at the G_1_ phase, modulated the progression of the S phase, and effectively restored the proliferative capacity of T cells. By synergistically blocking the PARP and PD-L1 dual signaling pathways, Awuah-3 exerted a robust antitumor effect.

### 5.4. PD-L1/JAK inhibitors

Janus kinases (JAK) are a non-receptor tyrosine kinase that regulates cytokine-mediated immune and inflammatory responses through the JAK-STAT signaling pathway, serving as therapeutic target for various autoimmune diseases and myeloproliferative disorders 161. Activation of the JAK-STAT signaling pathway can upregulate PD-L1 expression on tumor cells through cytokine-mediated mechanisms (e.g., IFN-γ), thereby contributing to the formation of an immunosuppressive microenvironment and exhibiting functional interplay with the PD-1/PD-L1 immune checkpoint pathway 162, 163. Wang *et al.* synthesized the PD-L1/JAK inhibitor PJ27 (PD-1/PD-L1: IC_50_ = 414 nM, JAK1: IC_50_ = 786 nM) (42, Figure 8; Table 4) 164. In the LLC lung cancer mouse model, PJ27 exhibited a dose-dependent tumor growth inhibitory effect: at 50 mg/kg, its achieved a TGI rate of 56%, significantly outperforming the single-target PD-L1 inhibitor BMS-202 at the same dose. Meanwhile, PJ27 could effectively downregulate PD-L1 expression in tumor tissues and inhibited STAT3 phosphorylation. It also markedly promoted the infiltration of CD3⁺CD8⁺ cytotoxic T cells and CD3⁺CD4⁺ helper T cells into the TME, thereby enhancing antitumor immune response. In addition, no significant decrease in the body weight of mice was observed during the administration period, and there were no notable pathological damages to major organs such as the heart, liver, spleen, lungs, and kidneys.

### 5.5. PD-L1/CD47 inhibitors

CD47-mediated signaling inhibits macrophage-mediated phagocytosis 165, 166. Collective inhibition of the PD-L1 and CD47 signaling pathways potentiated the tumor-suppressive effect in a synergistic manner 167, 168. Jin *et al.* developed SMC18 (**43**, Figure 8; Table 4), a bispecific inhibitor targeting both the CD47/SIRPα (IC_50_ = 17.8 μM) and PD-1/PD-L1 (IC_50_ = 19.7 μM) signaling axes 168. *In vitro*, 50 μM SMC18 showed no obvious cytotoxicity to tumor cells. It significantly inhibited the tyrosine phosphorylation of SIRPα and PD-1, effectively restore the phagocytic capacity of bone marrow-derived macrophages against MC38 and B16-OVA tumor cells, and meanwhile recover the IL-2 secretion Jurkat cells. *In vivo*, in the MC38 colorectal cancer-bearing mouse model, SMC18 as a single agent could significantly inhibit tumor growth, effectively promote the infiltration of CD8⁺ T cells and M1-type macrophages into tumor sites, and enhance the IFN-γ secretion capacity of CD8⁺ T cells in the TME. Moreover, SMC18 at these doses caused no obvious hematotoxicity or hepatotoxicity, and no pathological damage was observed in the major organs of mice. In addition, the combination of SMC18 with local radiotherapy exerted a significant synergistic antitumor effect, which further inhibited tumor growth without exerting adverse effects on the body weight of mice.

### 5.6. PD-L1/CD73 inhibitors

CD73 functions as an ecto-5'-nucleotidasethat converts extracellular AMP into adenosine, a potent immunosuppressive metabolite 169. Combining CD73 suppression with PD-L1 inhibition can counteract adenosine-mediated immune suppression and enhance tumor control 170, 171. Wang *et al.* covalently linked BMS-1233 (PD-L1 inhibitor) with LY-3475070 (CD73 inhibitor) to construct a series of bifunctional compounds, among which CC-5 (PD-L1: IC_50_ = 6 nM; CD73: IC_50_ = 773 nM) (**44**, Figure 8; Table 4) exhibited the best activity 172. At the cellular level, CC-5 exhibited no significant toxicity to PBMCs and CT26 tumor cells, yet dose-dependently enhanced PBMCs-mediated killing activity of CT26 cells. Meanwhile, it effectively inhibited the PD-1/PD-L1 interaction, exerting immunomodulatory effects. *In vivo*, CC-5 showed remarkable tumor growth inhibitory activity in both the CT26 colorectal cancer and B16-F10 melanoma model. Additionally, CC-5 could significantly elevate the infiltration level of CD3⁺CD8⁺ cells in tumor tissues, effectively activating the TME and thereby exerting a synergistic antitumor effect.

### 5.7. PD-L1/EGFR inhibitors

EGFR is frequently overexpressed or mutated in epithelial malignancies 173. EGFR signaling mediates PD-L1 overexpression in malignant cells 174. Yang *et al.* integrated the biphenyl/pyridine core of BMS-202 with the quinazoline scaffold of gefitinib (EGFR inhibitor) to synthesize a series of bifunctional compounds, among which EP26 (**45**, Figure 8; Table 4) emerged as the most promising candidate 175. EP26 exhibited inhibitory activity against both EGFR (IC_50_ = 37.5 nM) and PD-1/PD-L1 (IC_50_ = 1.77 μM). *In vitro*, Its antiproliferative effects across various glioblastoma (GBM) cell lines significantly surpassed that of control drugs such as Gefitinib. Mechanistically, EP26 induced G_0_/G_1_ phase cell cycle arrest, downregulated EGFR phosphorylation, and reactivated immunosuppressed T cells. Notably, EP26 possessed favorable pharmacokinetic properties with an oral bioavailability of 22.2% and blood-brain barrier penetration ability. *In vivo*, in GBM mouse model, EP26 administered at 100 mg/kg achieved a TGI rate of 92.0%, outperforming Gefitinib and NP19 (PD-L1 inhibitor), without obvious toxicity. Although EP26 increased the proportions of CD4⁺ and CD8⁺ cells in the TME, the increase was significantly lower than that of NP19. It exerted antitumor effects through the dual mechanisms of direct tumor inhibition and immune activation.

### 5.8. PD-L1/Tubulin inhibitors

Microtubules play essential role in cellular motility, division, and intracellular trafficking, while additionally impacting immune cell responses 176. Concomitant inhibition of PD-L1 and microtubules exhibited synergistic antitumor efficacy 177. Yang *et al.* adopted a hybridization strategy, integrated the key structural fragments of these two classes of inhibitors, and designed and synthesized 20 CA-4 analogs, among which TP5 (**46**, Figure 8; Table 4) exhibited the most potent activity 178. *In vitro*, TP5 exhibited potent inhibitory effects on five cancer cell lines, including HepG2 and MC38, and paclitaxel-resistant A549/PTX cells, with an IC_50_ value of 0.8 μM in HepG2 cells and extremely low cytotoxicity to normal cells. TP5 inhibited tubulin polymerization (IC_50_ = 16.10 μM), disrupted the microtubule network, arrested the cell cycle at the G_2_/M phase, induced apoptosis, and suppressed tumor cell migration and colony formation. Additionally, TP5 moderately inhibited the PD-1/PD-L1 interaction (IC_50_ = 48.76 μM), showing similar binding affinities to both human and murine PD-L1. *In vivo*, intragastric administration of TP5 at 100 mg/kg achieved TGI rate of 57.9% in the humanized PD-1 melanoma mouse model, without significant hepatotoxicity, nephrotoxicity, cardiotoxicity, or myelosuppression. Meanwhile, TP5 upregulated the mRNA expression of CXCR3 and CXCL10, downregulated PD-L1 expression in tumor tissues, increased the proportion of tumor-infiltrating T cells, and activated the TME.

### 5.9. PD-L1/CXCL12 inhibitors

The chemokine CXCL12 (SDF-1) contributes to immunosuppression in the TME by recruiting PD-L1-expressing cells and promoting tumor cell survival and metastasis 179. Dual inhibition of the PD-L1 and CXCL12 pathways enhances antitumor activity by mitigating these immunosuppressive mechanisms 180. Chen *et al.* synthesized bifunctional compounds by conjugated the pharmacophores of PD-L1/CXCL12 inhibitors via a linker, among which CP21 (**47**, Figure 8; Table 4) exhibited the optimal activity 181. CP21 embedded into the inner cavity of the PD-L1 dimer and bound to PD-L1 through hydrophobic interactions (IC_50_ = 78.6 nM), while it could bind to the hydrophobic cleft of CXCL12 (K_D_ = 160 nM), thus exhibiting a stable binding mode. Pharmacokinetic studies showed that CP21 had a higher plasma exposure and a lower clearance rate upon intravenous administration, and also exhibited acceptable plasma exposure following oral dosing. *In vivo*, in B16-F10 melanoma and CT-26 colon cancer models, CP21 inhibited tumor growth in a dose-dependent manner with a better efficacy than monotherapy, and it also significantly increased the proportions of CD3⁺ and CD3⁺CD8⁺ T cells, as well as the CD8⁺/CD4⁺ ratio in tumor tissues, effectively activating the TME. Meanwhile, CP21 caused no obvious hepatic, renal or cardiac toxicity and induced no morphological abnormalities in tissues, exhibiting a good safety profile.

### 5.10. PD-L1/VISTA inhibitors

V-domain Ig suppressor of T-cell activation (VISTA), a PD-1 homolog and immune checkpoint, exhibited therapeutic synergy when co-blocked with PD-L1 182, 183. CA-170 (**48**, Figure 8; Table 4) was the first oral PD-L1/VISTA inhibitor to enter clinical trials (completed phase I and advanced to phase II) 184. However, its binding mechanism was controversial. On the one hand, nuclear magnetic resonance (NMR) and homogeneous time-resolved fluorescence (HTRF) assays verify that it does not directly bind to PD-L1, as its core Ser-Asn-Thr (SNT) motif was derived from the BC loop of AUNP-12—a region distant from the PD-1/PD-L1 interface—thus failing to achieve specific binding. However, this did not negate the clinical potential of CA-170. It was speculated that its antitumor mechanism did not directly block the PD-1/PD-L1 interaction; instead, it might have acted on the downstream signaling molecules of the PD-1 pathway and regulated other T cell activation pathways 185. On the other hand, cellular NMR experiments suggested that it bound to PD-L1 in cells without prevent the assembly of the PD-1/PD-L1 complex, but instead formed a functionally defective ternary complex 186. Preclinical monotherapy or combination therapy with docetaxel and cyclophosphamide could effectively inhibit tumor growth or reduce metastasis and exhibited good safety. Adverse reactions were mainly mild to moderate, such as fatigue and nausea. The core advantages were convenient oral administration, dual-target synergy, and high production cost potential. However, there were still issues such as unclear binding mechanisms, narrow dose windows, and small sample sizes in some clinical data remain to be resolved 184, 187.

Wang *et al.* identified the benzo[d]oxazole derivative B3 (**49**, Figure 8; Table 4) as a dual inhibitor of PD-L1/VISTA 188. This compound inhibited the PD-1/PD-L1 interaction (IC_50_ = 26.2 nM) and bound to VISTA (K_D_ = 107 nM). Moreover, B3 induced the degradation of both PD-L1 and VISTA through the autophagy-lysosome pathway. *In vitro*, it restored T-cell function and enhanced the antitumor immunity of PBMCs. *In vivo*, treatment with B3 suppressed tumor growth in a dose-dependent manner in the CT26 mouse model, showing superior efficacy to positive drugs and their combination therapy. Additionally, it increased CD8⁺ T cell infiltration into tumors.

Sun *et al.* adopted a scaffold fusion strategy, in which the biphenyl structure of BMS-202 and the ethanolamine-pyrimidine ring structure of the VISTA inhibitor A4, were fused to synthesize a series of compounds. Among these, P17 (**50**, Figure 8; Table 4) was identified as the most promising candidate 189. P17 induced PD-L1 dimerization (IC_50_ = 149.2 nM) and bound to VISTA (K_D_ = 272.3 nM), thereby blocking its interaction with endogenous ligands. At the cellular level, P17 exhibited no significant cytotoxicity, concentration-dependently enhanced PBMCs antitumor activity, promoted IFN-γ secretion, and relieved VISTA-mediated T cell suppression. *In vivo*, its antitumor efficacy surpassed that of PD-L1 or VISTA single-target inhibitors. Additionally, P17 significantly increased the infiltration and activation of CD8^+^ T cells, downregulated the proportion of Tregs, and optimized TME. Moreover, it showed good safety with an oral bioavailability of 11.86%.

In the field of dual inhibitors of PD-L1 and other targets, a “pharmacophore hybridization” or “skeleton fusion” strategy was adopted for drug design (e.g., PD-L1/HDAC inhibitors, PD-L1/EGFR inhibitors, PD-L1/VISTA inhibitors). This approach leveraged well-defined immune synergistic mechanisms between targets (e.g., pathway complementarity and upstream-downstream regulation), solid preclinical data (e.g., tumor suppressive activity across multiple tumor models, favorable safety profiles, and partially optimized pharmacokinetic characteristics), and improved drug-like properties, with some agents (e.g., the PD-L1/VISTA inhibitor CA-170) having entered clinical validation. However, these bifunctional molecules face multiple core challenges in practical research and development. Achieving a balanced potency against both targets simultaneously proved difficult. Structurally, their increased complexity often led to suboptimal pharmacokinetic profiles, including low oral bioavailability and inadequate half-lives. The multi-domain design also raised the risk of off-target effects. Moreover, the majority of these inhibitors remained in the preclinical stage, lacking support from large-scale clinical data. Insufficiently in-depth research on synergistic mechanisms and drug resistance, as well as the lack of clear predictive biomarkers for efficacy, further limit their clinical application and development.

## 6. Conclusions and perspectives

Malignant tumor cells have demonstrated the ability to circumvent immune surveillance through a range of immunosuppressive mechanisms, and ICT has revolutionized the clinical landscape of cancer treatment 2, 3, 5. The PD-1/PD-L1 axis is recognized as a central molecular mechanism underlying tumor immune escape 9. Although the U.S. FDA has authorized multiple anti-PD-1/PD-L1 mAbs, these agents were limited by their macromolecular properties and efficacy bottlenecks. These challenges have steered the development of small molecule immuno-oncology agents toward three core strategies: inhibitors, degraders, and dual-target inhibitors. Although inhibitors and degraders share similar functions in inhibiting the PD-1/PD-L1 axis, they exert fundamentally distinct effects on immune homeostasis and present distinct profiles of irAEs. PD-L1 inhibition function by reversibly blocking the PD-1/PD-L1 interaction 190. However, they are often insufficient to target the entire cellular PD-L1 pool. The residual PD-L1 retains partial physiological immune regulatory functions, causing mild and reversible disturbance to immune homeostasis with lower risk of mostly mild-to-moderate irAEs 191. In contrast, PD-L1 degraders irreversibly eliminates the protein through the ubiquitin-proteasome or lysosomal pathway. This approach not only completely blocks the pro-tumor immunosuppressive function of PD-L1 but also abrogates its physiological role in maintaining immune tolerance, resulting to a more profound and sustained disturbance to immune homeostasis and a higher risk of irAEs 192. However, the application of targeted delivery systems can enhance tumor-specific accumulation to reduce off-target effects, and the more thoroughly blockade of PD-L1-mediated pro-tumor signals (including ligand-independent ones) by degraders may offer more durable therapeutic efficacy 193. From a biological standpoint, PD-L1 inhibition and degradation may result in different degrees of immune modulation, and careful consideration of immune homeostasis and safety will be important for future clinical translation.

Most PD-L1 inhibitors utilize a biphenyl core scaffold that adapts to the hydrophobic binding cavity of PD-L1 through rotation, inducing dimerization / endocytosis. For example, CCX559 67, 68, S4-1 83, INCB086550^73^, BMS-202^88^-^90^. Some have adopted C2-symmetric or dual-site binding structures, such as GS-4224 47 and CB31 53, to bind two PD-L1 molecules. To overcome single-action limitations or optimize pharmacokinetics, heterocycles 94, metal ligands 86, and natural product scaffolds 79 have been incorporated, whereas terminal hydrophilic groups address the poor water solubility of pure aromatic structures 72, 95. PD-L1 degraders employ a “ligand/carrier-linker-effector ligand” modular design: As heterobifunctional entities, PROTACs incorporate a PD-L1-binding ligand, a specific E3 ubiquitin ligase ligand, and a connecting linker segment—linker properties regulate the formation of the ternary complex 122, 124, 125, while LYTACs target lysosomes via LTR ligands with linkers maintaining dual-ligand activity; for example, CI-M6PR ligands 129-131, scavenger receptor ligands 134, cRGD 135, cell-penetrating peptides 137. Dual-target inhibitors integrate PD-L1 active moieties (predominantly biphenyl scaffolds) with secondary target pharmacophores via “pharmacophore hybridization”. For example, hydroxamate for HDAC6/3 150, 153, indolinone for IDO1 156, benzimidazole for CD47 168, furopyrimidine for CD73 172, quinazoline for EGFR 175, with linkers of tailored length/rigidity/hydrophilic-lipophilic balance. Also adopted the “scaffold fusion” strategy to synergistically modulate the PD-1/PD-L1 pathway and another key target, balancing dual-target efficacy and drug-likeness 185, 188.

The research and development of PD-1/PD-L1 inhibitors has reached a key and sophisticated development stage 194. Although small molecule inhibitors offer distinct advantages such as oral bioavailability, low immunogenicity, and strong tumor penetration, antibody-based therapies remains the mainstream clinical practice, with small molecule mostly serving as complementary options 18, 195. Because small molecule inhibitors face multiple core bottlenecks in clinical translation. First, in terms of drug resistance, primary and adaptive resistance coexist with heterogeneous mechanisms, making preemptive intervention difficult. Second, insufficient target specificity predisposes these agents to off-target toxicity, which may disrupt normal immune homeostasis or induce organ damage 196. Third, pharmacokinetics is affected by factors such as individual metabolic differences and short half-lives, which limits the practical advantages of oral administration 195. Fourth, clinical validation mostly remains in the early stages, lacking large-scale phase II data for direct comparison with antibody therapies. For instance, published clinical data for INCB086550 have been suboptimal, and its phase II results remain undisclosed; moreover, predictive biomarkers for efficacy are not yet well defined. Fifth, tumor heterogeneity renders them difficult to adapt to different PD-L1 expression types and cancer types 197. Finally, issues such as purity control and stability during production also constitute hidden challenges 198. To address these limitations, future development of PD-1/PD-L1 inhibitors will mainly focus on three key strategic directions: optimization of combination regimens, epigenetic targeting, and development of novel drug modalities 199. combination strategy focuses on “resistance and insufficient target binding”, epigenetic targeting directly addresses “abnormal target expression regulation and heterogeneity”, and novel drug modalities optimize around “safety, selectivity, and clinical translation efficiency”. In terms of drug combination strategies, compensatory activation of alternative targets and synergistic efficacy enhancement can be achieved through combination with other immunomodulatory targets and chemotherapy/radiotherapy/anti-angiogenic agents 8. At the epigenetic targeting level, aimed at addressing abnormalities in PD-1/PD-L1 methylation, histone deacetylation, and chromatin remodeling, specific regulatory drugs can be developed 200. With respect to advancing emerging drug modalities, next-generation small molecule inhibitors, PROTACs/LYTACs degraders, and bispecific antibodies will be leveraged to overcome the limitations of traditional antibodies, thereby improving targeting specificity and safety 17. With the advancement of mechanistic research and technological innovations, PD-1/PD-L1 inhibitors are poised to propel tumor immunotherapy toward greater precision and efficiency.

## Figures and Tables

**Table 1:** Comparison between PD-1/PD-L1 small molecule inhibitors and monoclonal antibodies

Feature	PD-1/PD-L1 monoclonal antibodies	PD-1/PD-L1 small molecule inhibitors	Advantage of small molecules
Molecular size	Large (~150 kDa)	Small (<1 kDa)	Easier tissue penetration
Target binding	Extracellular PD-1/PD-L1 blockade	Target PD-1/PD-L1 interaction or intracellular regulators	Broader targeting possibilities
Route of administration	Intravenous infusion	Often oral or injectable	More convenient administration
Pharmacokinetics	Long half-life (weeks)	Shorter half-life (hours-days)	Better dose control and flexibility
Tumor penetration	Limited in solid tumors	Improved diffusion in tumor microenvironment (TME)	Better access in dense tumor tissue
Manufacturing	Complex biologic production	Chemical synthesis, scalable	Lower production cost
Storage and stability	Requires cold chain	More stable, easier storage	Improved accessibility worldwide
Immunogenicity	Potential immune-related antibody responses	Generally low immunogenicity	Reduced risk of anti-drug antibodies
Immune-related adverse events	Often prolonged due to long persistence	Potentially reversible (shorter duration)	Safer management of toxicity
Target spectrum	Highly specific for PD-1/PD-L1	Can be dual-target or multi-pathway modulators	Expanded therapeutic scope
Intracellular accessibility	Cannot enter cells	Can modulate intracellular signaling molecules	Expanded druggable space
Combination potential	Limited by long exposure and toxicity overlap	Flexible combinational regimens	Improved adaptability
Cost	Very expensive	Potentially lower cost	Improved affordability

**Table 2:** Clinical small molecule PD-L1 inhibitors

Compd.	Inventor	Structure	Biological data	Clinical Trail	Ref.
GS-4224 (**1**)	Gilead Sciences	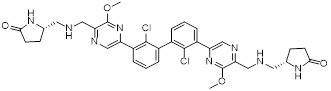	IC_50_ = 0.213 nM	NCT04049617, phase I; (Terminated)	65
CCX559 (**2**)	ChemoCentryx, Inc.	-	IC_50_ = 0.47 nM	ACTRN12621001342808, phase I	67, 68
ASC61 (**3**)	GannexPharma Co., Ltd.	-	-	NCT05287399, phase I	69
MAX-10181 (**4**)	Maxi novel Pty, Ltd.	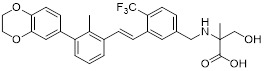	IC_50_ = 18 nM	NCT05196360, phase I; (Unknown status) NCT04122339, phase I; (Unknown status)	70
BPI-371153 (**5**)	Betta Pharmaceuticals Co, Ltd.	-	IC_50_ = 4.2 nM	NCT05341557, phase I (Recruiting)	72
INCB086550 (**6**)	Incyte Corporation	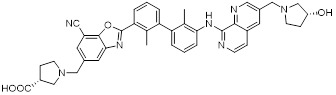	IC_50_ = 3.1 nM	NCT03762447, phase I; (Completed)、 NCT04674748, phase I; (Terminated)、 NCT04629339, phase II; (Terminated)、 NCT05101369, phase I; (Completed)	73, 74
YPD-29B (**7**)	Tianjin Chasesun Pharmaceutical Co., Ltd.	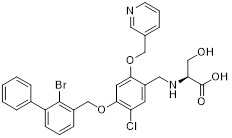	IC_50_ < 0.1 pM	NCT04343859, phase I; (Unknown status)	77

“-” means the data is unavailable (not public or non-existent).

**Table 3:** Preclinical small molecule PD-L1 inhibitors

Compd.	Inventor	Structure	Biological data	Ref.
MM-129 (**8**)	Medical University of Bialystok	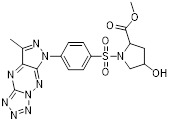	IC_50_ = 3.1 μM (DLD-1 cell)	79
S4-1 (**9**)	China Pharmaceutical University	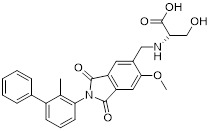	IC_50_ = 6.1 nM	83
CB31 (**10**)	Chulalongkorn University	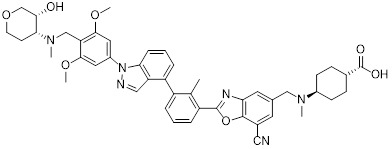	IC_50_ = 0.2 nM	84
APBC (**11**)	Lanzhou University	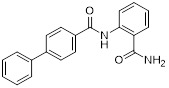	IC_50_ = 27.82 μM	85
Pt-2 (**12**)	Sun Yat-sen University	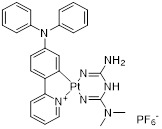	IC_50_ = 490 nM	86
SA-49 (**13**)	Chinese Academy of Medical Sciences	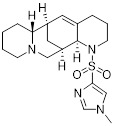	-	87
BMS-202 (**14**)	BMS	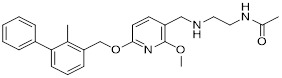	IC_50_ = 18 nM	88, 90
HD10 (**15**)	Zhejiang University of Technology	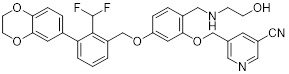	IC_50_ = 3.1 nM	93
Compound **16**	China Pharmaceutical University	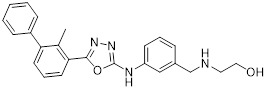	IC_50_ = 27.8 nM	94
NPH 16 (**17**)	China Pharmaceutical University Southern Medical University	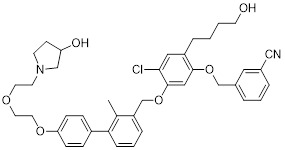	IC_50_ = 24.4 nM	95
D6 (**18**)	Capital Medical University	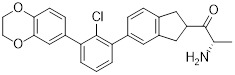	IC_50_ = 4.8 nM	96
ARB-272572(**19**)	Arbutus Biopharma Inc, Xtal BioStructures Inc.	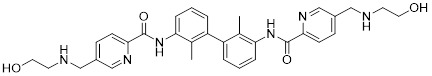	IC_50_ = 400 pM	97
Anidulafungin (**20**)	College of Pharmacy, Jiangsu University	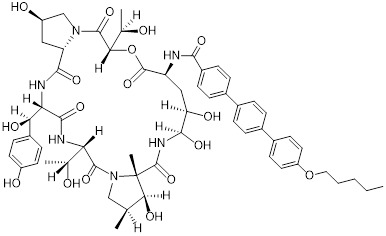	-	98
CBPA (**2**1)	Lanzhou University	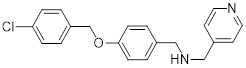	IC_50_ = 57.67 μM (MC38 cell) IC_50_ = 77.45 μM (B16F10 cell)	99

“-” means the data is unavailable (not public or non-existent).

**Table 4:** Co-inhibitors of PD-L1 and other targets

Compd.	Inventor	Target	Structure	Biological data	Ref.
Bian-10 (**38**)	China Pharmaceutical University	PD-L1/ HDAC6	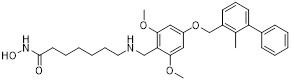	IC_50_ = 85 nM (PD-L1); IC_50_ = 23 nM (HDAC6)	150
PH3 (**39**)	Southern Medical University	PD-L1/ HDAC3	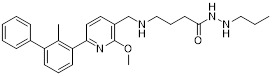	IC_50_ = 89.4 nM (PD-L1); IC_50_ = 107 nM (HDAC3)	153
Feng-1 (**40**)	Chinese Academy of Medical Sciences	PD-L1/ IDO1	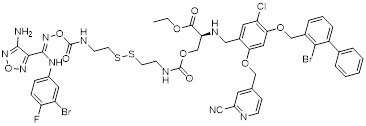	-	156
Awuah-3 (**41**)	University of Kentucky	PD-L1/ PARP	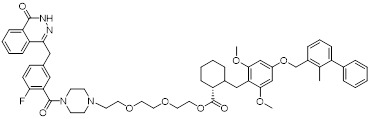	-	160
PJ27 (**42**)	Henan University of Chinese Medicine	PD-L1/ JAK	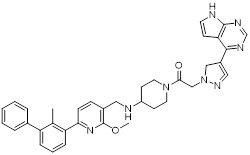	IC_50_ = 414 nM (PD-L1); IC_50_ = 786 nM (JAK)	164
SMC18 (**43**)	Zhengzhou University	PD-L1/ CD47	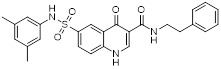	IC_50_ = 19.7 μM (PD-L1); IC_50_ =17.8 μM (CD47)	168
CC-5 (**44**)	Wenzhou Medical University	PD-L1/ CD73	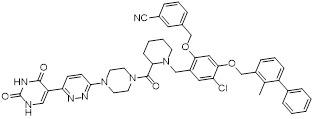	IC_50_ = 6 nM (PD-L1); IC_50_ = 773 nM (CD73)	172
EP26 (**45**)	Southern Medical University	PD-L1/ EGFR	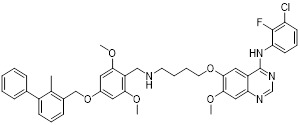	IC_50_ = 1.7 μM (PD-L1); IC_50_ = 37.5 nM (EGFR)	175
TP5 (**46**)	Southern Medical University	PD-L1/ Tubulin	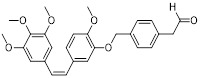	IC_50_ = 48.76 μM (PD-L1); IC_50_ = 16.10 μM (Tubulin)	178
CP21 (**47**)	Hubei Polytechnic University	PD-L1/ CXCL12	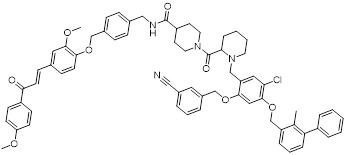	IC_50_ = 78.6 nM (PD-L1);	181
CA-170 (**48**)	Curis	PD-L1/ VISTA	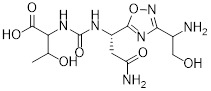	-	184, 186, 187
B3 (**49**)	China Pharmaceutical University	PD-L1/ VISTA	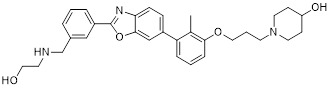	IC_50_ = 26.2 nM (PD-L1); K_D_ = 107 nM (VISTA)	188
P17 (**50**)	China Pharmaceutical University	PD-L1/ VISTA	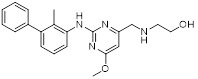	IC_50_ = 149.2 nM (PD-L1); K_D_ =272.3 nM (VISTA)	189

“-” means the data is unavailable (not public or non-existent).

## Abbreviations

mAbs: monoclonal antibodies
FDA: food and drug administration
PROTACs: proteolysis-targeting chimeras
LYTACs: lysosome-targeting chimeras
CTLA-4: cytotoxic T-lymphocyte-associated antigen 4
ICT: immune checkpoint therapy
PD-1: programmed cell death protein 1
PD-L1: programmed death-ligand 1
ITSM: immunoreceptor tyrosine-based switch motif
ITIM: immunoreceptor tyrosine-based inhibitory motif
TME: tumor microenvironment
NK cells: natural killer cells
DCs: dendritic cells
SH2: Src homology 2
SHP2: Src homology 2 domain-containing tyrosine phosphatase 2
TCR: T cell receptor
EGF: epidermal growth factor
IL-17: interleukin-17
TNF-α: tumor necrosis factor-alpha
LPS: lipopolysaccharide
IFN-γ: interferon-gamma
PTEN: phosphatase and tensin homolog
RAS: rat sarcoma virus
EGFR: epidermal growth factor receptor
PLCγ1: phospholipase C gamma 1
PIP_2_: phosphatidylinositol 4,5-bisphosphate
DAG: diacylglycerol
IP_3_: inositol trisphosphate
PKCθ: protein kinase Cθ
CBM: CARMA1-BCL10-MALT1
NSCLC: non-small cell lung carcinoma
DLBCL: diffuse large B-cell lymphoma
CLL: Chronic lymphocytic leukemia
PBL: plasmablastic lymphoma
HNSCC: head and neck squamous cell carcinoma
PDAC: pancreatic ductal adenocarcinoma
SBRT: stereotactic body radiation
TAMs: tumor-associated macrophages
ccRCC: clear cell renal cell carcinoma
CAR-T: chimeric antigen receptor T cells
CAIX: carbonic anhydrase IX
Tfh: T follicular helper cells
PBMCs: peripheral blood mononuclear cells
BCAT1: branched-chain amino acid transaminase 1
TGI: tumor growth inhibition rate
irAEs: immune-related adverse events
PK/PD: pharmacokinetic/pharmacodynamic
SAR: structure-activity relationships
sPD-L1: soluble programmed death-ligand 1
AbTACs: antibody-based PROTACs
EPR: enhanced permeability and retention
FMD: folic acid-modified dextran
NRP-1: neuropilin-1
LTR: lysosomal targeting receptor
CI-M6PR: cation-independent mannose-6-phosphate receptor
SRs: Scavenger receptors
cRGD: cyclic Arg-Gly-Asp
IFLDs: integrin-facilitated lysosomal degraders
HIP1R: huntingtin-interacting protein 1-related
CPPs: cell-penetrating peptides
HDAC: histone deacetylase
IDO1: indoleamine 2,3-dioxygenase 1
PARP: poly (ADP-ribose) polymerase
JAK: Janus kinase
EGFR: epidermal growth factor receptor
VISTA: V-domain Ig suppressor of T-cell activation
NMR: nuclear magnetic resonance
HTRF: homogeneous time-resolved fluorescence
SNT: Ser-Asn-Thr
